# The circRNA *circVAMP3* restricts influenza A virus replication by interfering with NP and NS1 proteins

**DOI:** 10.1371/journal.ppat.1011577

**Published:** 2023-08-21

**Authors:** Jie Min, Yucen Li, Xinda Li, Mingge Wang, Huizi Li, Yuhai Bi, Ping Xu, Wenjun Liu, Xin Ye, Jing Li

**Affiliations:** 1 CAS Key Laboratory of Pathogenic Microbiology and Immunology, Institute of Microbiology, Chinese Academy of Sciences, Beijing, China; 2 Savaid Medical School, University of Chinese Academy of Sciences, Beijing, China; 3 Department of Microbiology and Parasitology, College of Basic Medical Sciences, China Medical University, Shenyang, China; 4 School of Life Sciences, University of Science and Technology of China, Anhui, China; 5 Department of Microbiology and Immunology, Virginia Commonwealth University, Richmond, Virginia, United States of America; 6 Institute of Infectious Diseases, Shenzhen Bay Laboratory, Shenzhen, Guangdong, China; University of Texas Medical Branch / Galveston National Laboratory, UNITED STATES

## Abstract

Circular RNAs (circRNAs) are involved in various biological roles, including viral infection and antiviral immune responses. To identify influenza A virus (IAV) infection-related circRNAs, we compared the circRNA profiles of A549 cells upon IAV infection. We found that *circVAMP3* is substantially upregulated after IAV infection or interferon (IFN) stimulation. Furthermore, IAV and IFN-β induced the expression of QKI-5, which promoted the biogenesis of *circVAMP3*. Overexpression of *circVAMP3* inhibited IAV replication, while *circVAMP3* knockdown promoted viral replication, suggesting that *circVAMP3* restricts IAV replication. We verified the effect of *circVAMP3* on viral infection in mice and found that *circVAMP3* restricted IAV replication and pathogenesis *in vivo*. We also found that *circVAMP3* functions as a decoy to the viral proteins nucleoprotein (NP) and nonstructural protein 1 (NS1). Mechanistically, *circVAMP3* interfered with viral ribonucleoprotein complex activity by reducing the interaction of NP with polymerase basic 1, polymerase basic 2, or vRNA and restored the activation of IFN-β by alleviating the inhibitory effect of NS1 to RIG-I or TRIM25. Our study provides new insights into the roles of circRNAs, both in directly inhibiting virus replication and in restoring innate immunity against IAV infection.

## Author summary

Influenza A virus (IAV) poses a serious public health and socio-economic threat as a result of pandemic and seasonal epidemics. IAV utilizes host cell mechanisms to promote its replication, while the host innate immune system can induce a variety of antiviral agents to fight IAV infection. Recent studies have found that more and more noncoding RNAs (ncRNAs) are involved in antiviral immune response. Circular RNAs (circRNAs), a new member of ncRNAs, are involved in a variety of biological roles, and most studies have focused on their function in cancer. Recently, several research groups have found that circRNAs have significantly changed upon IAV infection and they also take part in viral infection and antiviral immune responses. However, the biogenetic processes, potential functions, and underlying mechanisms of most circRNAs in IAV infection remain unclear. Here, we screened the circRNA transcription profiles of three IAV strains to identify circRNAs involved in viral replication. We found that *circVAMP3* was also induced by IFN-β during IAV infection, with the largest increase. We further found that circ*VAMP3* formation is mediated by QKI-5, which acts as a decoy for viral proteins to inhibit IAV replication. Our study provides new insights into the role of circRNAs against IAV infection.

## Introduction

Influenza A viruses (IAVs), zoonotic pathogens that continuously circulate, pose a threat to public health and can cause human epidemics or pandemics [1,2]. IAV, which belongs to the *Orthomyxoviridae* family, consists of eight different negative-sense, single-stranded RNA segments encoding at least 17 proteins [3]. The virus ribonucleoprotein (vRNP) complex, which consists of viral RNA (vRNA), multiple nucleoproteins (NPs), and RNA polymerase complex polymerase basic 1 (PB1), PB2, and polymerase acidic protein (PA), plays essential roles in IAV genome replication and transcription [4,5]. The vRNP complex contains nuclear localization signal (NLS) peptides, which are recognized by the nuclear localization carrier protein importin-α in host cells, mediating entry of the vRNP complex into the nucleus. Once the viral proteins are synthesized in the cytoplasm, the self-dependent NLS is introduced into the nucleus, where vRNP complex assembly is completed [6–9]. Specifically, the viral nonstructural protein 1 (NS1) protein is widely recognized as a cofactor through which IAVs resist the host immune response [10].

The host innate immune system can be activated upon IAV infection [11]. In the process of viral infection, the antiviral immune response is triggered when the virus or its genetic material is detected by cellular pattern recognition receptors, followed by activating downstream signaling pathways to induce the expression of type I interferons (IFNs), such as IFN-β [12]. Type I IFNs promote the transcription of several genes called IFN-stimulating genes (ISGs). ISGs and other proteins with antiviral potential (restriction factors) can antagonize the viral life cycle. For example, IFN-inducible transmembrane proteins inhibit IAV entry by blocking the binding of viral envelope protein to the host membrane [13]. MxA functions as a broad-spectrum antiviral protein by capturing viral components in the early stage of infection and preventing the virus from entering cells [14]. ZAP and PKR inhibit IAV replication by interfering with the translation system [15,16]. Cyclophilin A interacts with the matrix protein (M1) of IAV and inhibits the transport of M1 into the nucleus [17]. Furthermore, noncoding RNAs (ncRNAs) are also involved in antiviral innate immunity. For instance, microRNA-33a inhibits IAV replication by targeting ARCN1 and repressing vRNP activity [18]. Lnc-ISG20 was found to disrupt the inhibitory effect of miR-326 on *ISG20* mRNA, followed by increasing the protein level of ISG20 to inhibit viral replication [19]. However, further elaboration on the underlying mechanisms of ncRNAs utilizing innate immune responses against IAV infection is required.

Circular RNAs (circRNAs) are a new class of ncRNAs that originate from nonsequential back splicing of exons and/or introns of precursor messenger RNAs (pre-mRNAs). CircRNAs were first discovered in RNA viruses in 1976 and described as single-stranded, covalently closed molecules [20]. Then, Hsu *et al*. found that endogenous circRNAs were also present in eukaryotic cells [21]. Subsequent studies have shown that circRNAs are widely distributed in various eukaryotes and exhibit highly conserved and tissue-specific characteristics [22]. Due to their circular conformation, circRNAs are more stable than mRNAs. CircRNAs interact with RNA-binding proteins (RBPs) or microRNAs (miRNAs) and thereby affecting their activities, regulating a variety of cellular processes, such as transcription and translation [23,24]. According to recent studies, circRNAs play important roles in innate immunity, suggesting that circRNAs have the potential to affect IAV replication. Indeed, Qu *et al*. found that the upregulation of the circRNA AIVR by IAV inhibits IAV replication by sponging miRNAs and promoting the expression of CREBBP to facilitate IFN-β production [25]. In addition, RBPs are essential for circRNA generation [26]. However, the biogenesis processes, underlying functions, and potential mechanisms of circRNAs in IAV infection remain elusive.

In this study, we screened the circRNA transcriptional profiles of A549 cells infected with different IAV H1N1 strains (WSN, PR8, or CA04) to identify several circRNAs involved in IAV replication. We found a set of circRNAs that were stimulated by IAV. We then focused on the circRNA with the greatest increase upon IAV infection, *circVAMP3*, as it is also induced by IFN-β and inhibits IAV replication. Further analysis indicated that *circVAMP3* biogenesis is mediated by QKI-5, which is upregulated by IAV and IFN-β. Mechanistically, *circVAMP3* serves as a decoy to the viral NP and NS1 proteins, which interfere with vRNP complex activity and inhibit the effect of NS1 on the RIG-I signaling pathway. Together, these findings revealed that *circVAMP3* plays an important role in restricting IAV replication.

## Results

### Both IAV infection and IFN-β stimulation upregulate *circVAMP3*

**Fig 1:**
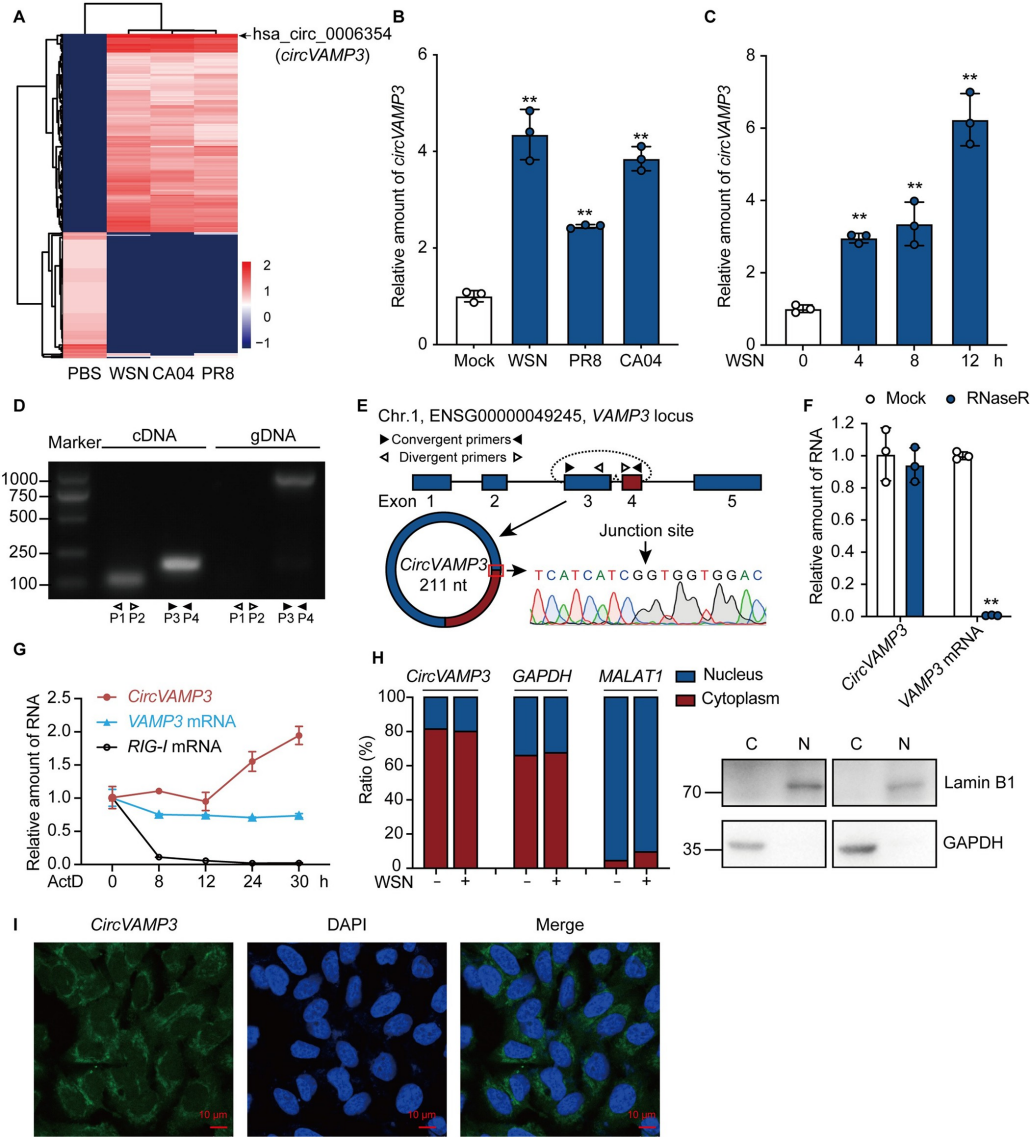
IAV infection enhances *circVAMP3* expression. (A-C) A549 cells were infected with IAV (WSN, CA04, or PR8) at an MOI of 1 for 8 h. (A, B) or infected with WSN for the indicated times (C). Total RNA was extracted, treated with RNase R, and analyzed using RNA sequencing (A) or RT-qPCR (B, C). (A) Clustered heat map showing circRNAs with a > 2-fold change in expression in cells infected with the three strains of IAV. Relative expression values are indicated based on color and increase in value from red to blue (log scale 2, from −1 to +2). The black arrow indicates the position of circular RNA VAMP3 (*circVAMP3*). (D) The presence of *circVAMP3* in A549 cells was validated by RT-PCR using divergent primers (P1 and P2) and convergent primers (P3 and P4) to detect VAMP3 and *circVAMP3* from cDNA or gDNA. (E) Schematic illustration showing the genomic location of *circVAMP3* generated from the back-splicing of exons 3 and 4 of the VAMP3 pre-mRNA and sequencing data. The blue arrow indicates the junction site of *circVAMP3*. (F, G) The relative expression levels of *circVAMP3*, VAMP3 (F), and RIG-I (G) in A549 cells with or without RNase R treatment (F) or with actinomycin D treatment (G) were quantified using RT-qPCR. (H) A549 cells were infected with or without WSN at an MOI of 0.5 for 8 h. RNA was extracted from nuclear and cytoplasmic fractions and subjected to RT-qPCR to detect *circVAMP3*, GAPDH, and MALAT1 (H, left panel). The fractionated cell lysates were immunoblotted with anti-GAPDH and anti-Lamin B1 antibodies (H, right panel). (I) A549 cells were fixed and subjected to RNA FISH with a biotin-labeled *circVAMP3* junction-specific antisense probe (green). Nuclei were stained with DAPI (blue). Scale bar, 10 μm. Data are presented as the means ± SD; *n =* 3; **p < 0.01. Data are representative of results from at least three independent experiments.

To identify circRNAs involved in IAV infection, A549 cells were infected with IAV H1N1 strains (WSN, PR8, or CA04), and circRNA screening was performed by deep RNA sequencing. We found differences in the expression levels of many circRNAs when cells infected with all three strains compared to the uninfected cells (S1A Fig). Among them, the levels of 260 circRNAs were significantly altered in all cells respectively infected with the three IAV strains (S1B Fig), in which 159 circRNAs upregulated and 101 circRNAs downregulated (Fig 1A). Among these circRNAs, *hsa_circ_0006354* was the most significantly upregulated upon IAV infection. We then validated that *hsa_circ_0006354* was upregulated in IAV-infected cells using RT-qPCR (Fig 1B and 1C). In addition, we examined whether *hsa_circ_0006354* expression is induced by other stimuli and found that *hsa_circ_0006354* is also upregulated by Sendai virus (SeV) (S1C Fig), poly (I:C) (S1D Fig), and IFN-β (S1E Fig).

To verify the presence of *hsa_circ_0006354*, the convergent and divergent primers were designed for cDNA and genomic DNA (gDNA) PCR amplification (Fig 1D). Sanger sequencing of the PCR product, the head-to-tail splice junction site amplified by the divergent primers, indicated that *hsa_circ_0006354* is derived from *VAMP3* gene exons 3 and 4, which located on human chromosome 1 (Fig 1E), and *hsa_circ_0006354* was accordingly named *circVAMP3*. *CircVAMP3*, except *VAMP3* mRNA, was resistant to RNase R treatment (Fig 1F) and had a longer half-life than *VAMP3* and *RIG-I* mRNAs (Fig 1G). Interestingly, we observed that *VAMP3* mRNA had a longer half-life than *RIG-I* mRNA (Fig 1G). We then examined the subcellular localization of *circVAMP3* by performing cell fractionation and RNA FISH. It revealed that *circVAMP3* is mainly located in the cytoplasm but at very low levels in the nucleus (Fig 1H and 1I). Together, *circVAMP3* is expressed at high levels upon IAV infection or IFN-β stimulation and is located mainly in the cytoplasm.

### QKI-5 promotes the generation of *circVAMP3*

**Fig 2:**
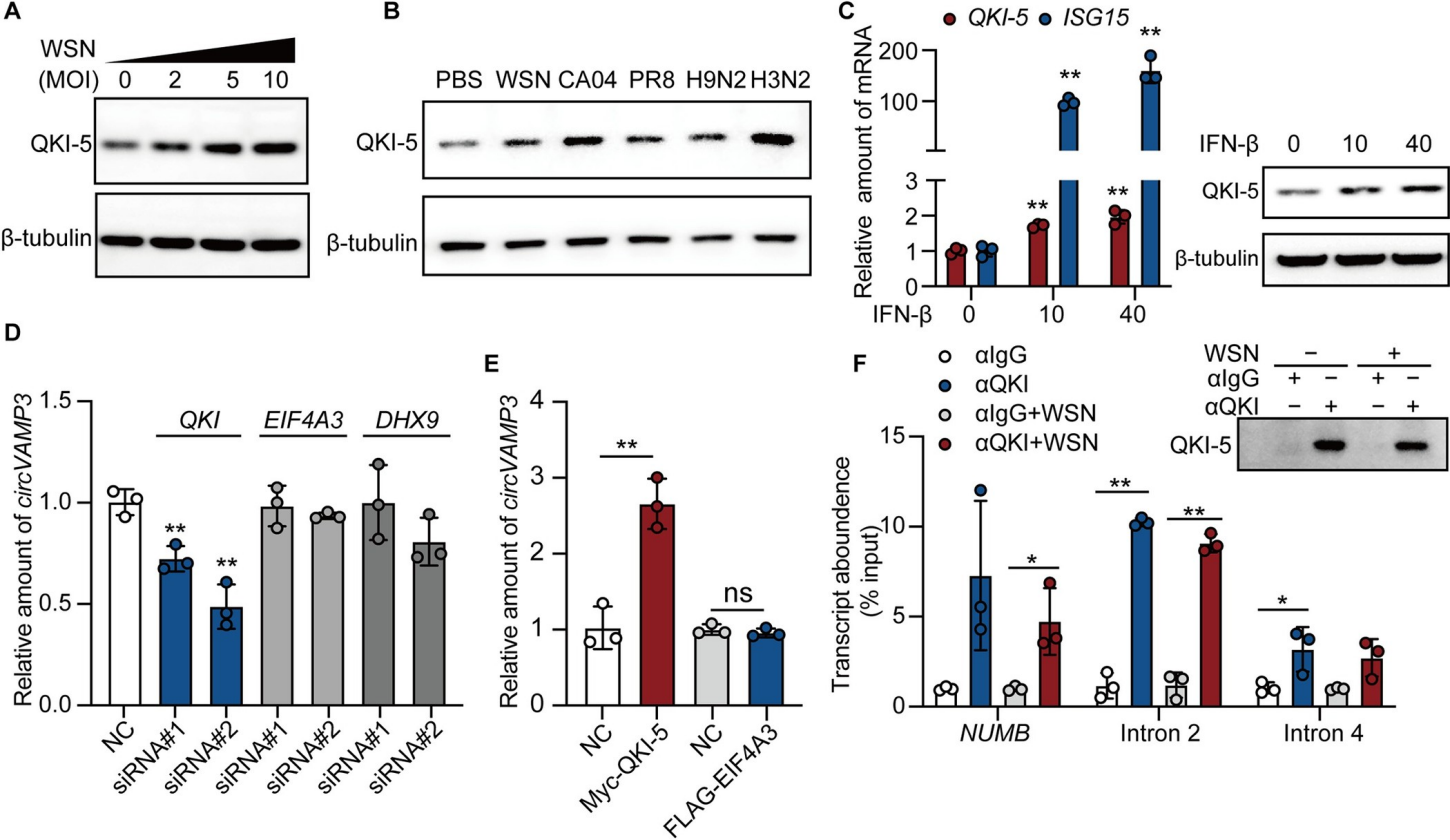
QKI-5 binds the flanking introns to facilitate the biogenesis of *circVAMP3*. (A) A549 cells were infected with WSN at an MOI of 0, 2, 5, or 10 for 4 h, and the cell lysates were collected for immunoblotting assays using the indicated antibodies. (B) A549 cells were treated with PBS or infected with IAV (WSN, CA04, PR8, H9N2, or H3N2) at an MOI = 5 for 4 h, and the cell lysates were harvested for immunoblotting assay. (C) A549 cells were directly stimulated with IFN-β at concentrations of 10 ng/mL and 40 ng/mL for 8 h. Cells were collected for RNA extraction to detect the transcription level of *QKI-5* and *ISG15* (as a positive control) (left), and cell lysates were collected to detect the protein level of QKI-5 (right). (D) Effect of QKI, EIF4A3, or DHX9 knockdown on the abundance of *circVAMP3* in A549 cells. (E) RT-qPCR detection of *circVAMP3* from 293T cells overexpressing QKI-5 or EIF4A3. Myc-EV or FLAG-EV served as the control. (F) A549 cells were infected with or without WSN at an MOI of 0.5 for 2 h. (F, left panel) RIP assay using intron 2 or intron 4 PCR primers. The validated QKI binding site in NUMB was used as a positive control. Cell lysates were immunoprecipitated with an anti-QKI antibody or IgG as the control. (F, right panel) The pulled-down QKI-5 protein was detected by immunoblotting with an anti-QKI-5 antibody. The data shown in C, D, E, and F are presented as the means ± SD; *n =* 3; *p < 0.05, **p < 0.01. Data are representative of the results from at least three independent experiments.

It is known that splicing factors play a role in regulating the biogenesis of circRNAs [27]. To explore the mechanism by which *circVAMP3* is formed, we identified two and two putative binding sites for EIF4A3 and DHX9, respectively, in the flanking region of *circVAMP3* using Circinteractome (https://circinteractome.nia.nih.gov) and found three binding site motifs of ACUAAY (Y  =  C or U) for QKI-5 (S2A Fig). We hypothesized that if the three proteins contribute to *circVAMP3* biogenesis, they would also be regulated by IAV. To test this hypothesis, we also explored the process of *circVAMP3* biogenesis upon H1N1 WSN infection by conducting a proteomics analysis of A549 cells in the early stage of WSN infection. Among the eight circRNA biogenesis-associated proteins, only QKI-5 was significantly upregulated upon WSN infection (S2B Fig). We confirmed that the levels of QKI-5 expression and transcription were significantly upregulated after WSN infection, while the levels of EIF4A3 and DHX9 did not change (S2C and S2D Fig). Next, to explore whether IAV regulation of QKI-5 is viral titer dose-dependent, we used multiplicities of infection (MOIs) with different gradients to stimulate A549 cells and cell lysates were collected 4 h after infection for western blotting. We found that compared to the uninfected group, the protein level of QKI-5 increased gradually with the increase of virus titer (Fig 2A), indicating that H1N1 WSN upregulates *circVAMP3* cycling-related protein QKI-5 in a dose-dependent manner. To investigate the effect of other IAV strains on QKI-5 expression. A549 cells were infected with the three types of H1N1 (WSN, CA04, and PR8), the avian IAV H9N2, and H3N2, individually. Consistent with WSN infection, all viruses can significantly upregulate the protein level of QKI-5 (Fig 2B). Because both IAV and IFN-β upregulate *circVAMP3* expression, we hypothesized that IFN also influences QKI-5 transcription and protein levels. To test this hypothesis, A549 cells were incubated with IFN-β for 8 h, which significantly upregulated QKI-5 transcription and protein levels in a dose-dependent manner (Fig 2C). In conclusion, IAV can induce a significant increase in QKI-5 transcription and protein levels by up-regulating the expression of IFN.

Next, we examined the effects of QKI-5, EIF4A3, and DHX9 on the biogenesis of *circVAMP3*. We transfected A549 cells with two specific siRNAs targeting QKI-5, EIF4A3, or DHX9 or with scrambled siRNA for 48 h, Western blotting analysis showed that specific siRNAs treatment indeed downregulated the expression of QKI-5, EIF4A3, and DHX9 (S2E Fig). The expression level of *circVAMP3* was then measured in siRNA-treated cells. We found that QKI-5 knockdown significantly decreased *circVAMP3* generation, while EIF4A3 and DHX9 knockdown did not (Fig 2D). Then, we transfected QKI-5 or EIF4A3 (negative control) in 293T cells for 48 h to detect their effects on *circVAMP3* levels (S2F Fig). We found that the overexpression of QKI-5, not EIF4A3, increased *circVAMP3* expression (Fig 2E). It reported that QKI acts as a dimer capable of binding two separated pre-mRNA regions and facilitating the circle-forming exons proximity, followed to circRNAs biogenesis [28,29]. To assess whether QKI binds the VAMP3 pre-mRNA, we performed RNA immunoprecipitation (RIP) assays. The result demonstrated that QKI bound to the exon-adjacent sites of *VAMP3* pre-mRNA at a level comparable to NUMB, a binding site in the previously validated QKI target [30], no matter with or without IAV infection (Fig 2F). Together, these results suggest that QKI-5 expression induced by IAV infection and IFN-β stimulation promotes *circVAMP3* generation.

### *CircVAMP3* restricts IAV replication *in vitro*

To investigate the effect of *circVAMP3* on IAV replication, the pLC5-ciR-GFP plasmid was used as the backbone to insert the cyclization splicing site and the third and fourth exon sequences of VAMP3 between the *Eco*RI and *Bam*HI restriction sites. And then, 293T cells were transfected with pLC5-*circVAMP3* or pLC5-ciR-GFP (control). The *circVAMP3* was overexpressed in 293T cells (S3A Fig), and then the cells were infected with WSN virus to further explore the function of *circVAMP3* during IAV replication. The result showed that *circVAMP3* overexpression substantially reduced the viral titer (S3B Fig) and the level of the viral proteins M1 and NP (S3C Fig). However, we also found that the plasmid creates an additional 10 kDa fragment detected using VAMP3 antibody (S3C Fig). To eliminate the interference of this by-product, the *circVAMP3* junction site was redesigned in this study (Fig S3D), and we verified that 10-kDa protein was no longer produced (S3E Fig).

**Fig 3:**
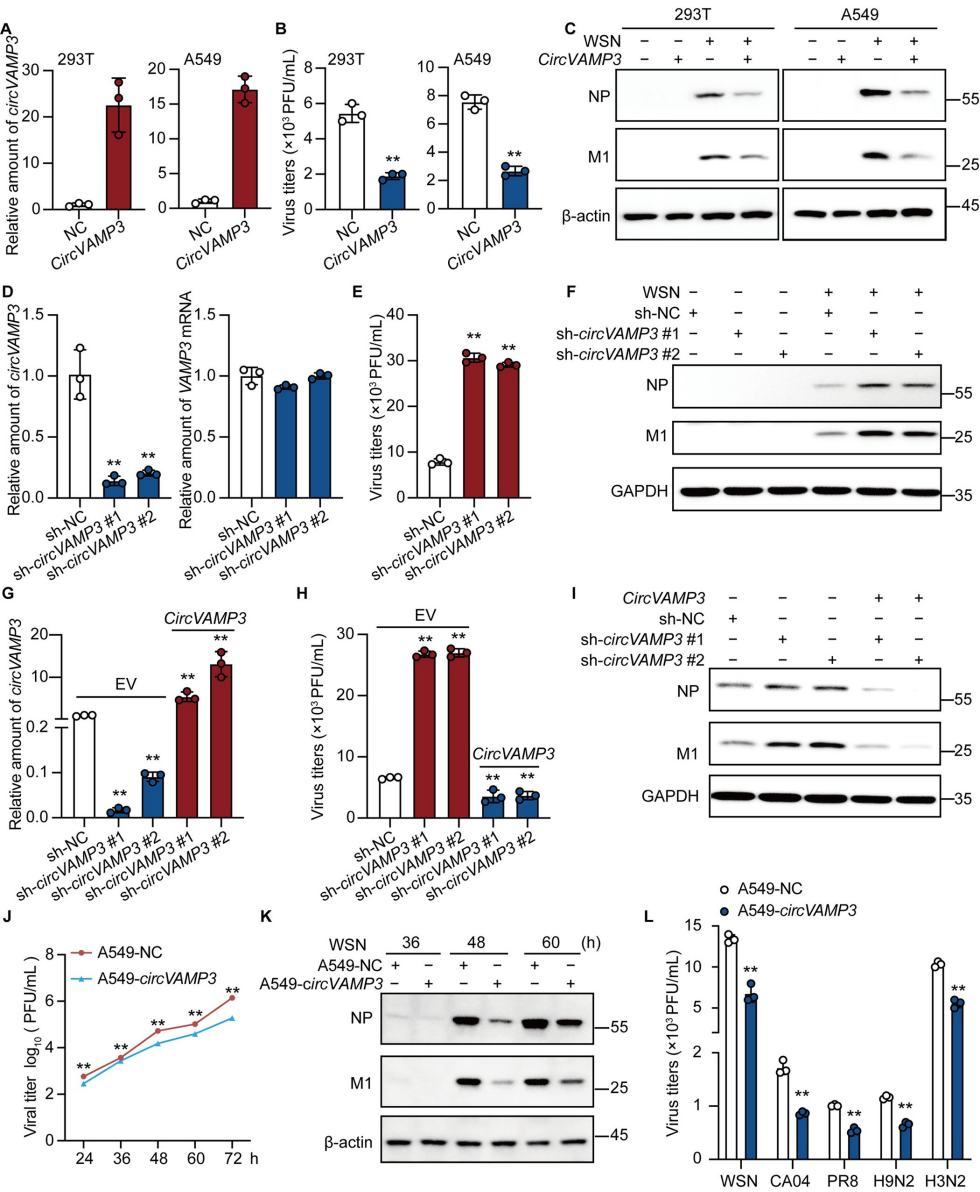
Overexpression of *circVAMP3* inhibits IAV replication, and *circVAMP3* knockdown enhances IAV replication in vitro. (A, B, and C) The 293T and A549 stable cell lines constructed were spread into 12-well plates for 18 h. And then infected with WSN at an MOI of 0.5 or treated with PBS for 8 h. (A) RT-qPCR confirmed that 293T and A549 stably overexpressed *circVAMP3*. (B) The supernatants of infected cells were collected for plaque tests to determine virus titer. (C) Cell lysates were collected and used for immunoblotting with corresponding antibodies. (D, E, and F) An A549 cell line with stable *circVAMP3* knockdown was constructed, and cells constructed with sh-NC-GFP no-load lentivirus were used as a control. After infection with *circVAMP3* lentivirus or control lentivirus for 72 h, A549 cells were plated in 12-well plates. The next day, the cells were infected with WSN at an MOI of 0.5 or treated with PBS. Cell supernatant and cell lysate were collected at 8 h. (D) 72 h after lentivirus infection, total RNA was collected for knockdown efficiency and off-target rate detection. (E) The cell supernatants were collected for plaque assays to determine virus titer. (F) The cell lysates collected were used for immunoblotting assays and incubated with the antibodies shown. (G, H, and I) Lip3000 was used to transfect pLC5-*circVAMP3* or pLC5-ciR-GFP (as negative control) in an A549 cell line with a stable knockdown of *circVAMP3*, and the sh-NC-GFP stable cell line was used to transfect pLC5-ciR-GFP in the control group. Twenty-four hours after transfection of corresponding plasmids, cells were infected with WSN at an MOI of 0.5, and cell supernatants and cell lysates were collected at 8 h. (G) RT-qPCR confirmed that the level of *circVAMP3*. (H) The cell supernatants collected were used for plaque assays to determine virus titer. (I) The cell lysates collected were used for immunoblotting assays and incubated with the antibodies shown. (J and K) The cells were grown in the 12-well plates and then infected with WSN (MOI = 0.01) without supplement TPCK-treated trypsin, the supernatants were followed harvested at the indicated time points for plaque assays to detect the viral growth curves (J), and the cell lysates were collected for immunoblotting assay (K). (L) The A549-NC and A549-*circVAMP3* cells were infected with IAV (WSN, CA04, PR8, H9N2, or H3N2) at an MOI = 0.5 for 16 h, and the cell supernatants were collected for plaque assays. Data shown in A, B, D, E, G, H, J, and L are presented as the mean ± SD. *n =* 3; **p < 0.01. These data represent the results of at least three independent experiments.

To identify the function of *circVAMP3* during IAV replication, we generated the 293T-*circVAMP3* and A549-*circVAMP3* cell lines, which stably ectopically expresses *circVAMP3*, and the control cell lines 293T-NC and A549-NC (Fig 3A). To verify the overexpression efficiency, 293T and A549 cells were infected with pLC5-*circVAMP3* lentivirus particles, and stable cell lines were constructed. Seventy-two hours later, the infected cells were collected, cell RNA was extracted for reverse primer amplification, the amplified products were purified, and they were sequenced. The amplification products from cells infected with pLC5-*circVAMP3* lentivirus were more intense than those infected with empty lentivirus (S3F Fig), and the sequencing results were consistent with the expected new cyclic interface sequence (S3F Fig). The established cells were then infected with WSN virus. The results demonstrated that stable *circVAMP3* overexpression substantially reduced the viral titer (Fig 3B) and the level of the viral proteins M1 and NP (Fig 3C). Consistent results were observed in the two *circVAMP3*-overexpressing stable cell lines, suggesting that *circVAMP3* inhibits IAV replication. To verify whether the effect of *circVAMP3* on WSN replication was dose-dependent, 293T cells were transfected according to the doses shown in S3G Fig, cell lysates were collected under the same conditions as above, and western blotting was performed. We found that transfection of 500 ng could significantly reduce the NP and M1 protein levels of WSN, and with the gradual increase of *circVAMP3* transfection dose, the NP and M1 protein levels gradually decreased. These results indicate that transient *circVAMP3* overexpression in 293T cells can significantly inhibit the replication of IAV in a dose-dependent manner.

To further explore the effect of endogenous *circVAMP3* on IAV replication, we stably knocked down *circVAMP3* in A549 cells using lentivirus-mediated short hairpin RNAs (shRNAs) targeting two different junction sites of *circVAMP3*, causing a reduction in *circVAMP3* expression but not *VAMP3* mRNA levels (Fig 3D), and then infected the cells with WSN. We observed that *circVAMP3* depletion significantly increased the viral titer in (Fig 3E), as well as the levels of both the M1 and NP proteins (Fig 3F). To further demonstrate that the observed phenotype was not due to off-target effects, we transfected pLC5-ciR-GFP (EV, control) or pLC5-*circVAMP3* into the two A549 stable knock-down cell lines, and then infected with WSN virus. As shown in Fig 3G, *circVAMP3* levels were ~10-fold higher than control cells. We found that the *circVAMP3* restoration group significantly decreased the titer of WSN virus in A549 cells (Fig 3H), as well as the level of M1/NP protein (Fig 3I), compared to the empty plasmid. Taken together, these results suggest that *circVAMP3* affects IAV replication in different cell types.

To further explore the effect of *circVAMP3* at later time points. Therefore, we tested WSN replication in A549-*circVAMP3* cells in comparison to that in A549-NC cells. The samples were harvested at 24 h, 36 h, 48 h, 60 h, and 72 h after infection. The result demonstrated that the virus titer of A549-*circVAMP3* cells exhibited obvious reduction at every indicated time point (Fig 3J). Especially, the titer exhibited an approximately 0.9 log reduction at 72 hp.i. (Fig 3J). Meanwhile, the cell lysates were harvested for the Western blotting assay, and *circVAMP3* can significantly reduce the NP and M1 protein levels of WSN at later time points compared to control cells. To determine the function of *circVAMP3* in cells infected with other subtype IAVs, we infected A549-NC and A549-*circVAMP3* with WSN, CA04, PR8, H9N2, and H3N2, respectively. The supernatants were collected for plaque assay and the data showed that the viral titer of WSN, CA04, H9N2, and H3N2 from A549-*circVAMP3* cells was significantly lower than that from control cells (Fig 3L). These results demonstrated that *circVAMP3* can strongly inhibit the replication and proliferation of WSN at multiple longer time points and can inhibit the proliferation of multiple strains of IAV.

Given that VAMP3 is highly homologous in human and mice, mouse ortholog Vamp3 also has the two exons of *circVAMP3*. However, *circVamp3* doesn’t exist in mice through searching from circBase (http://www.circbase.org/). To verify no homologous *circVamp3* in mice, we designed the divergent and convergent primers to amplify the *circVamp3*, *circIpo11*(positive control, which was verified to exist in mice), and Vamp3 mRNA in Lewis lung carcinoma (LLC1) cell lines of C57BL mice and the lung of BLAB/c. The data showed that neither in mice cell line no matter whether infected with IAV (S4A Fig) nor in mice lung (S4B Fig) exist *circVamp3*. And then, we investigated the level of Qki-5 after WSN infected LLC1 cell line. The data showed that the transcription level of Qki-5 in mice cells was not significantly different upon WSN infection (S4C Fig). To quantify the direct contribution of *circVAMP3* expression in mice cells, we generated LLC1 cells harboring stable *circVAMP3* expression vectors (LLC1-*circVAMP3*) or control vectors (LLC1-NC) (S4D Fig), and then infected cells with WSN. Supernatants and cell lysates were collected for plaque assay and immunoblotting, respectively. The data showed that the viral titer of the supernatant from LLC1-*circVAMP3* cells was significantly lower than that in LLC1-NC cells (S4E Fig), and levels of the viral protein M1 and NP were much lower in LLC1-*circVAMP3* cells than in LLC1-NC cells (S4F Fig). Taken together, no homologous *circVamp3* existed in mice, but overexpressed the *circVAMP3* of human still can inhibit WSN replication and proliferation in mice cell line.

Collectively, these results demonstrated that *circVAMP3* plays an important role in IAV replication.

### *CircVAMP3* restricts IAV replication and pathogenesis *in vivo*

**Fig 4:**
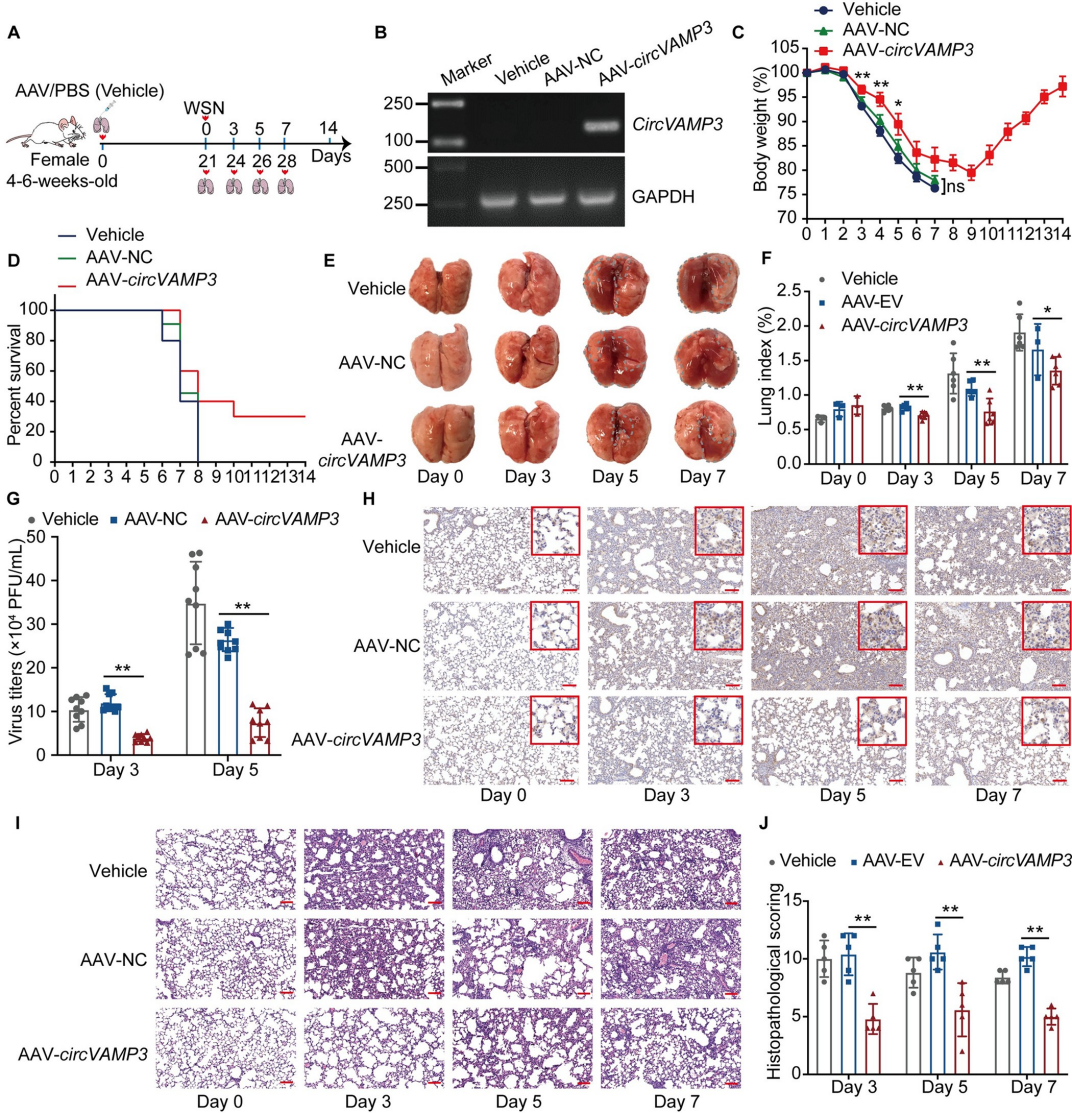
Overexpression of *circVAMP3* inhibits IAV replication in *vivo*. (A) Experimental design. (B to J) An adeno-associated virus (serotype 6, AAV6) carrying *circVAMP3* (AAV6-*circVAMP3*) or EV (AAV6-NC) was generated. BALB/c (4–6 weeks old, female) mice were administered PBS, AAV6-NC, or AAV6-*circVAMP3* in a volume of 50 μL (5×10^10^ vector genomes) through intratracheal instill. Then, the lungs of the mice were harvested at 3 weeks post-infection for RT-PCR analysis of exogenous *circVAMP3* expression (B). The mice were infected with WSN (10^4^ PFU/each) by nasal drip. The body weight (C) and survival rate (D) of the mice were monitored daily for 14 days (*n =* 10). At 0, 3, 5, and 7 dpi, a necropsy was performed (E), the gray dashed curves indicated the significant gross pathology and hemorrhage, and the lungs of the infected mice were collected and used to measure the lung index (*n =* 3 or 6) (F). At 3 and 5 dpi, the lungs of the infected mice (*n =* 3) were harvested for plaque assays (*n* = 3) to measure the viral titers (G) and the lung tissues were stained with anti-M1 mAb for immunohistochemistry (H) or with hematoxylin and eosin (*n =* 2), the red line indicated 100 μm (I). (J) Pathological scores were obtained by evaluating the pulmonary interstitial edema, alveolar edema, inflammatory invasion, and alveolar hemorrhage areas and degree of hyaline membrane development (*n =* 5). The data shown in C, F, G, and J are presented as the means ± SD; *p < 0.05 and **p < 0.01.

To investigate the effect of *circVAMP3* on IAV infection *in vivo*, AAV6-expressing *circVAMP3* (AAV6-*circVAMP3*) or negative control AAV6 (AAV6-NC) were delivered into the lungs of BALB/c mice by intratracheal intubation, with PBS serving as a control (Fig 4A). RT-PCR confirmed that exogenous *circVAMP3* was expressed in the lungs of the mice at 3 weeks post-infection (Fig 4B). We then examined the effect of *circVAMP3* on viral pathogenicity in the mice. We intranasally infected BALB/c mice with WSN and measured their body weights and daily survival. The data showed that the mice treated with AAV6-*circVAMP3* lost less body weight than the mice treated with AAV6-NC or PBS (Fig 4C and S1 Table). Additionally, the mortality rate of the AAV6-*circVAMP3*-treated mice was lower than that of the mice treated with AAV-NC or PBS. The AAV6-NC- and PBS-treated mice died within 8 dpi, while 30% of the AAV6-*circVAMP3*-treated mice were still alive at the end of the experiment (Fig 4D). As shown in Fig 4E, the lungs of the AAV6-NC- or PBS-treated mice were characterized by severe red blood cell extravasation at 5 and 7 dpi, but this symptom was alleviated in the AAV6-*circVAMP3*-treated mice. The lung index (lung weight/body weight) of the AAV6-*circVAMP3*-treated mice was lower than that of the NC mice, suggesting that *circVAMP3* reduced pulmonary edema (Fig 4F). To explore the viral load in the lungs, the viral titers in the lungs of the mice at 3 and 5 dpi were examined by plaque assay and immunohistochemistry (IHC) staining of the lung was performed at 0, 3, 5, and 7 dpi using anti-M1 mAb. The viral titer in the lungs of the AAV6-*circVAMP3*-treated mice was significantly lower than that in the AAV-NC- or PBS-treated mice at 3 and 5 dpi (Fig 4G). Less positive signals were distributed in lung tissues of AAV6-*circVAMP3*-treated mice than that in the AAV-NC- or PBS-treated mice at 3, 5, and 7 dpi (Fig 4H), indicating a lower viral load in the AAV6-*circVAMP3*-treated mice. These results indicate that *circVAMP3* can inhibit the viral replication in the lungs of infected mice, thus having the potential to increase the survival of mice during IAV infection *in vivo*. Histological analysis of the lungs indicated that alveolar damage and interstitial inflammatory cell infiltration were less serious in the AAV6-*circVAMP3*-treated mice than those in the AAV6-NC- or PBS-treated mice at 3, 5, and 7 dpi (Fig 4J). Consistently, the histopathological scores for the lungs of the AAV6-*circVAMP3*-treated mice were significantly lower than those for the lungs of the control mice (Fig 4K). Taken together, these results suggest that *circVAMP3* reduces IAV replication and pathogenesis *in vivo*.

### *CircVAMP3* directly interacts with both IAV NP and NS1

**Fig 5:**
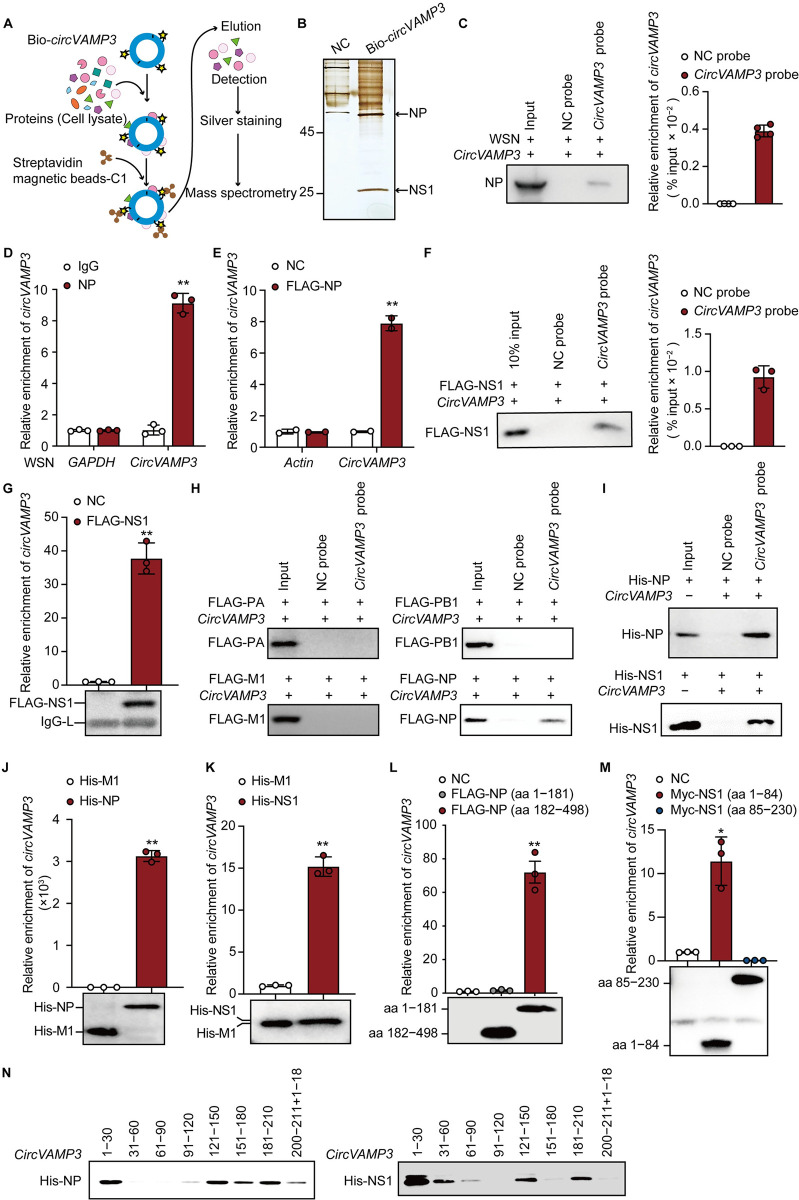
*CircVAMP3* directly binds to NP and NS1 of IAV. (A) Schematic of the biotin-labeled *circVAMP3* RNA pull-down assay. (B to D) A549 cells were infected with WSN at an MOI of 1 for 16 h. (B) The cell lysates were harvested and subjected to a pulldown assay with biotin-labeled *circVAMP3* or unlabeled *circVAMP3* as the control. The bound proteins were visualized by silver staining. The arrows indicate the bands that were subjected to mass spectrometry analysis. (C) The bound protein was detected by immunoblotting with anti-NP antibody (C, left). RT-qPCR was used to detect the efficiency of the pulldown with the probe (C, right) (*n* = 4). (D) The cell lysates were immunoprecipitated with anti-NP antibody or IgG as the control. (E) Cell lysates were harvested for immunoprecipitation with FLAG beads and subjected to RT-qPCR to detect *circVAMP3* or actin as an internal control (*n* = 2). (F) The bound NS1 protein was detected using immunoblotting. (G) The cell lysates were immunoprecipitated with FLAG beads. The bound NS1 was detected by immunoblotting (left panel). The bound *circVAMP3* was quantified using RT-qPCR (right panel). (H) The *circVAMP3* pull-down proteins were detected by immunoblotting with an anti-FLAG antibody. (I) The bound His-NP protein (upper panel) and His-NS1 (lower panel) were detected using immunoblotting with an anti-His antibody. (J) The bound *circVAMP3* was quantified by RT-qPCR (upper panel). His-NP and His-M1 were detected using immunoblotting with an anti-His antibody (lower panel). (K) The bound *circVAMP3* was quantified using RT-qPCR (upper panel). Pulled down His-NS1 and His-M1 were detected using immunoblotting with an anti-His antibody (lower panel). (L) The amount of bound *circVAMP3* was quantified using RT-qPCR (upper panel). The truncated NP proteins were detected using immunoblotting with an anti-FLAG antibody (lower panel). (M) The cell lysates were subjected to RNA immunoprecipitation with Myc beads. The bound *circVAMP3* was quantified using RT-qPCR (upper panel). The truncated NS1 proteins were detected using immunoblotting with an anti-Myc antibody (lower panel). (N) Truncated fragments of biotinylated *circVAMP3* (20 pmol) were incubated with His-NP (10 pmol) or NS1 (10 pmol). The bound NP or NS1 proteins were detected using immunoblotting with an anti-His antibody. The data shown in D, F, G, and J to M are presented as the means ± SD; *n =* 3; **p < 0.01. Data are representative of the results from at least three independent experiments.

We prepared biotin-labeled *circVAMP3* and performed an RNA pulldown assay, followed by mass spectrometry to identify *circVAMP3*-binding proteins and elucidate the mechanism by which *circVAMP3* inhibits IAV replication (Figs 5A, S5A and S5B). The result displayed that *circVAMP3* only interacts with the viral NP and NS1 proteins (Fig 5B). We then used RNA pulldown and RIP assays to further confirm whether *circVAMP3* interacts with viral NP and NS1. The biotin-labeled probe was incubated with the lysates of WSN-infected A549 cells for RNA pulldown to verify this result. As shown in Fig 5C, endogenous *circVAMP3*, which was specifically enriched by the probe specific for *circVAMP3*, could interact with NP. Then, following RNA immunoprecipitation, the precipitates were used for RT-qPCR-mediated detection of *circVAMP3* or *GAPDH* as an internal control. The results suggested the presence of endogenous *circVAMP3* in the viral NP-immunoprecipitated fraction (Fig 5D). We transfected 293T cells with pcDNA3-FLAG-NP or empty vector as a control to further confirm the interaction between *circVAMP3* and NP. We observed the presence of *circVAMP3* in the FLAG-immunoprecipitated fraction of FLAG-NP (Fig 5E). To characterize the interaction of *circVAMP3* with NS1, we transfected 293T cells with pcDNA3-FLAG-NS1 or an empty vector as a control. The cell lysates were harvested for RIP assays with a biotin-labeled *circVAMP3* probe or a random probe as control. As shown in Fig 5F, NS1 was pulled down with the *circVAMP3* probe, indicating that NS1 can interact with *circVAMP3*. Then, an RNA immunoprecipitation assay was performed, and RT-PCR was used to identify *circVAMP3*. The data showed the presence of *circVAMP3* in the FLAG-immunoprecipitated fraction of FLAG-NS1 (Fig 5G). Moreover, to verify *circVAMP3* does not bind to any IAV other proteins, we further transfected 293T cells with pcDNA-FLAG-PA, pcDNA-FLAG-PB1, pcDNA-FLAG-M1(as a negative control), and cDNA-FLAG-NP (as a positive control), respectively. The cell lysates were collected for RIP assays with a biotin-labeled *circVAMP3* probe or a random probe as control. As shown in Fig 5H, among the four viral proteins, only NP was pulled down with the *circVAMP3*, indicating *circVAMP3* does only interact with viral NP and NS1.

Next, we predicted the modeling structure using HADDOCK 2.4 and I-TASSER algorithm, which indicated that *circVAMP3* can directly interact with NP and NS1 by generating structural predictions (S5C and S5D Fig). To confirm whether *circVAMP3* directly interacts with NP or NS1 protein, we purified His-tagged NP, His-tagged NS1, and His-tagged M1 (as a negative control) from *Escherichia coli* and prepared *in vitro*-transcribed and self-circularized *circVAMP3*. We incubated His-NP/His-NS1 and *circVAMP3* with the biotin-labeled *circVAMP3* probe or a random probe as a control and then subjected the samples to pulldown with streptavidin beads, followed by immunoblotting with anti-NP or anti-NS1 antibody. This revealed the presence of NP or NS1 was both detected in the biotin-labeled *circVAMP3* probe precipitated fraction (Fig 5I). In addition, we performed immunoprecipitation with His-beads, followed by RT-qPCR to detect *circVAMP3* in the His-immunoprecipitated fraction. The presence of *circVAMP3* was detected in the His-immunoprecipitated fraction of His-NP or His-NS1 but not in the M1-immunoprecipitated fraction (Fig 5J and 5K), which further confirmed that *circVAMP3* can directly interact with NP or NS1 protein.

To determine the regions of NP responsible for interaction with *circVAMP3*, two truncated NP proteins based on the location of the RNA-binding domain (RBD) were expressed and subjected to RIP assays. Interestingly, the RT-qPCR results indicated that *circVAMP3* is associated with NP truncation mutant (aa 182–498) but not NP (aa 1–181) (the RBD) (Fig 5L). Two truncated NS1 proteins, based on the location of the RBP, were expressed and subjected to RIP assays to determine the exact domain of NS1 that interacts with *circVAMP3*. As expected, the RT-qPCR results indicated an interaction of *circVAMP3* with NS1 truncation mutant (aa 1–84) (the RBD) but not NS1 (aa 85–230) (Fig 5M). In addition, the four segments 1–30, 121–150, 151–180, and 181–210 nt of *circVAMP3* were sufficient to bind NP (Fig 5N). It demonstrated that *circVAMP3* directly interacts with the NP (aa 182–498) region and contains multiple NP-binding sites. In addition, the four segments 1–30, 31–60, 121–150, and 181–210 nt of *circVAMP3* were sufficient to bind NS1 (Fig 5N), indicating that *circVAMP3* shares partial interaction binding sites with NP and NS1. Taken together, these results indicate that *circVAMP3* directly interacts with NS1 (aa 1–84) region and contains multiple NS1-binding sites.

### *CircVAMP3* inhibits vRNP activity by impairing the interaction of NP with PB1 or PB2

**Fig 6:**
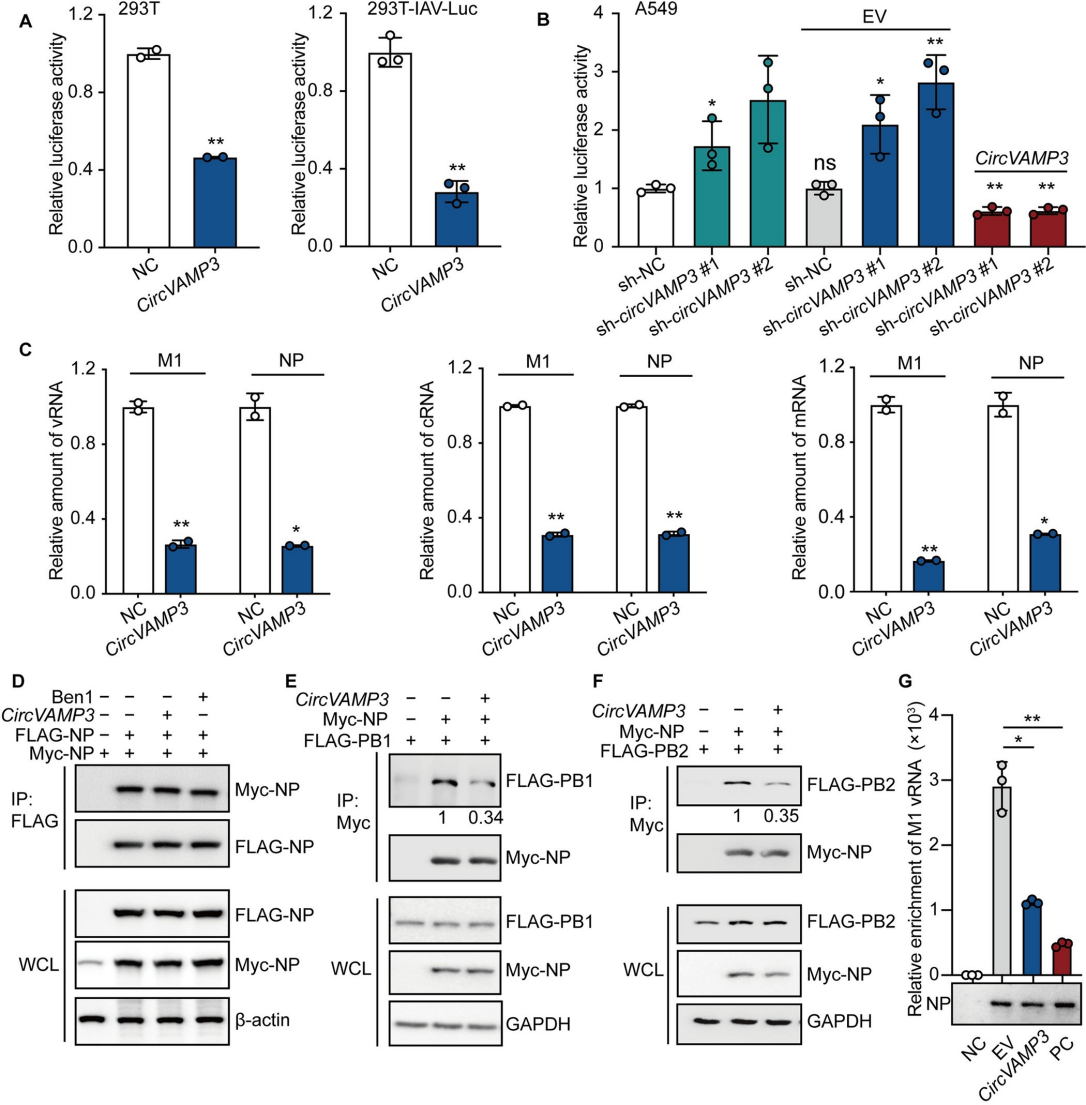
Overexpression of *circVAMP3* reduces viral RNAs transcription and replication by inhibiting the activity of the vRNP complex. (A, left) 293T cells were transfected with expression plasmids encoding PB1, PB2, PA, and NP of A/WSN/1933, vNS-Luc, and Renilla with the *circVAMP3* expression plasmid or empty vector (EV). The cell lysates were harvested for luciferase assays, and the results were normalized against Renilla luciferase activity (*n* = 2). (A, right) In 293T-IAV-Luc cells, the vRNP promoter-reporter (vNP-Luc) was stably expressed, and they were transfected with expression plasmids encoding PB1, PB2, PA, and NP of A/WSN/1933 with the *circVAMP3* expression plasmid or EV. The cell lysates were harvested for luciferase assays (*n* = 3). (B) The A549 stable knockdown cell line cells were transiently transfected with the four vRNP plasmids, vNS-Luc, and Renilla with EV/*circVAMP3*. (C) 293T cells were transfected with the *circVAMP3* expression plasmid or EV and then infected with A/WSN/1933 at an MOI of 1 for 8 h. Total RNA was extracted, and the vRNA (left), cRNA (middle), and mRNA (right) levels of M1 and NP were quantified by RT-qPCR (*n* = 2). (D) Cell lysates were immunoprecipitated with FLAG beads and immunoblotted with the indicated antibodies, using the WSN package plasmid ben1(PB2-vRNA) as a negative control. (E and F) The cell lysates were immunoprecipitated with Myc beads and immunoblotted with the indicated antibodies. (G) 293T cells were transfected with ben7-M-vRNA and Myc-NP/Myc-EV (as a negative control) with EV/*circVAMP3/*ben1-PB2-vRNA. The cell lysates were immunoprecipitated with Myc beads. And then immunoblotted with an anti-Myc antibody (lower panel) or the amount of bound ben7-M-vRNA (upper panel) was quantified using RT-qPCR (upper panel), the ben 1-PB2-vRNA as a positive control. The data shown in B and G are presented as the means ± SD; *n =* 3 **p < 0.01. Data are representative of the results from at least three independent experiments.

NP is an important component of the vRNP complex and is critical for vRNP activity [31–33]. We hypothesized that vRNP activity could be alleviated through the expression *circVAMP3* decoying NP. To test our hypothesis, we checked whether *circVAMP3* affects viral polymerase activity by interacting with NP. We performed a luciferase assay in 293T transfected with the vRNP promoter-reporter (driven by NS promoter), or in 293T-IAV-Luc cells (vRNP reporter stable expressed in 293T cells, driven by NP promoter) to observe the vRNP activity. As shown in Fig 6A, relative luciferase activity was significantly decreased in *circVAMP3*-overexpressing cells compared to the NC control, suggesting that *circVAMP3* inhibits viral polymerase activity. We then utilized the two types of A549-*circVAMP3* stable knock-down cells for the luciferase assays. We found that the vRNP activity was increased when the expression of *circVAMP3* was depleted, indicating that endogenous *circVAMP3* inhibits the transcription and replication of the viral genome, while *circVAMP3* reconstitution rescued the inhibition phenomenon (Fig 6B). To further determine the steps of viral transcription and replication that are inhibited by *circVAMP3* expression, we measured the vRNA, cRNA, and mRNA levels of M1 and NP in WSN-infected (8 h) 293T cells with or without *circVAMP3* overexpression. We found that *circVAMP3* overexpression reduces the vRNA, cRNA, and mRNA levels of M1 and NP (Fig 6C). Then, we utilized WSN-infected (4 h) A549-*circVAMP3* stable overexpression cells to measure the vRNA, cRNA, and mRNA levels of M1 and NP at the early stage of infection. The three RNA levels were all significantly reduced (S6A Fig). Furthermore, we analyzed the three RNA levels of M1 in the 293T *circVAMP3*-overexpressing cells infected with WSN for 2 h and 4 h. Interestingly, the M1 vRNA level was not significantly changed, but mRNA and cRNA were significantly decreased at 2 hp.i. (S6B Fig). All three RNA levels were significantly reduced at 4 hp.i (S6B Fig). These results indicated that *circVAMP3* overexpression can significantly inhibit the synthesis of vRNA, cRNA, and mRNA but had no significant effect on the entry of vRNA into host cells. In conclusion, *circVAMP3* impairs vRNP complex activity and thus reduces vRNA, cRNA, and mRNA synthesis.

Furthermore, we suspected that the vRNP activity was impaired due to the retention of vRNP and NP in cytoplasm caused by *circVAMP3* expression. Therefore, we explored whether *circVAMP3* has an inhibitory effect on the nuclear import of NP with a cell fractionation assay. The pLC5-*circVMAP3* or pLC5-ciR-GFP were transfected in 293T cells, followed infected with WSN at an MOI of 5. The cells were then lysed, and the nuclear and cytoplasmic fractions were separated and subjected to western blotting. As shown in S6C Fig, the marker proteins Lamin B1 and GAPDH were only detected in the nucleus and cytoplasm, respectively. At 1 and 2 hp.i., NP levels were not significantly changed in either the nucleus or cytoplasm of the *circVAMP3*-overexpressing cells compared to control cells. However, at 3 and 4 hp.i., NP was significantly downregulated in both the nucleus and cytoplasm of the *circVAMP3*-overexpressing cells compared to the control cells. These results suggested that *circVAMP3* indeed inhibits IAV replication at an early stage, but it can not affect incoming vRNP and NP nuclear import and virus entry. To further verify whether *circVAMP3* affects viral attachment or entry, we measured the effect of *circVAMP3* on viral attachment to cell membranes. As shown in S6D Fig, there was no significant difference in the amounts of infectious virus attached on the surface between the A549-*circVAMP3* cells and A549-NC cells. Meanwhile, we also harvested the cells for immunoblotting assay. As shown in S6E Fig, overexpression *circVAMP3* did not adversely affect the attachment of WSN to the A549 cell surface. We then verified whether overexpressed *circVAMP3* influences the internalization of IAV. The amount of internalized viral M1 protein between A549-*circVAMP3* and A549-NC has a minimal difference (S6F Fig). Taken together, *circVAMP3* inhibits IAV replication at the first replication cycle by impairing the activity of the vRNP complex, but *circVAMP3* is not required for IAV attachment to the cell surface and the internalization of IAV into cells.

To explore the molecular mechanism by which *circVAMP3* regulates IAV vRNP activity, we detected the effect of *circVAMP3* on impairing the function of NP during vRNP complex assembling. Previous studies report that NP aa 182–498 contains the NP self-polymerization region [34] and PB1 or PB2 binding region [33,35]. Since NP self-polymerization is important for vRNP activity, we wondered whether *circVAMP3* affects NP self-polymerization. We co-transfected 293T cells with pcDNA-FLAG-NP and pcDNA-Myc-NP along with the pLC5-*circVAMP3* or IAV-PB2-vRNA (Ben1, as a negative control). The cell lysates were then harvested for immunoprecipitation and immunoblotting. We found that *circVAMP3* did not influence NP self-polymerization (Fig 6D). Next, we sought to determine whether *circVAMP3* interfered with the interaction of NP and other subunits of the vRNP complex. To this end, we co-transfected 293T cells with pcDNA-Myc-NP, pcDNA-FLAG-PB1 or pcDNA-FLAG-PB2, and pLC5-*circVAMP3*. The cell lysates were harvested for immunoprecipitation and immunoblotting to detect the association of NP with PB1 or PB2. The overexpression of *circVAMP3* reduced the interaction of NP with PB1 or PB2 (Fig 6E and 6F). Meanwhile, we investigated whether *circVAMP3* influence NP interact with vRNA. We co-transfected 293T cells with pcDNA-Myc-NP, IAV-M-vRNA (Ben7), and pLC5-*circVAMP3 or* IAV-PB2-vRNA (Ben1, as positive control), the cell lysates were collected for Myc-immunoprecipitation. The samples were harvested for immunoblotting and RT-qPCR detection, respectively. The data showed that overexpression of *circVAMP3* reduced the interaction of NP with vRNA (Fig 6G). Taken together, *circVAMP3* inhibits IAV polymerase activity by interfering with the interaction of NP with other vRNP subunits.

### *CircVAMP3* alleviates the inhibitory effect of NS1 on IFN-β activation antagonizing NS1 binding to RIG-I

**Fig 7:**
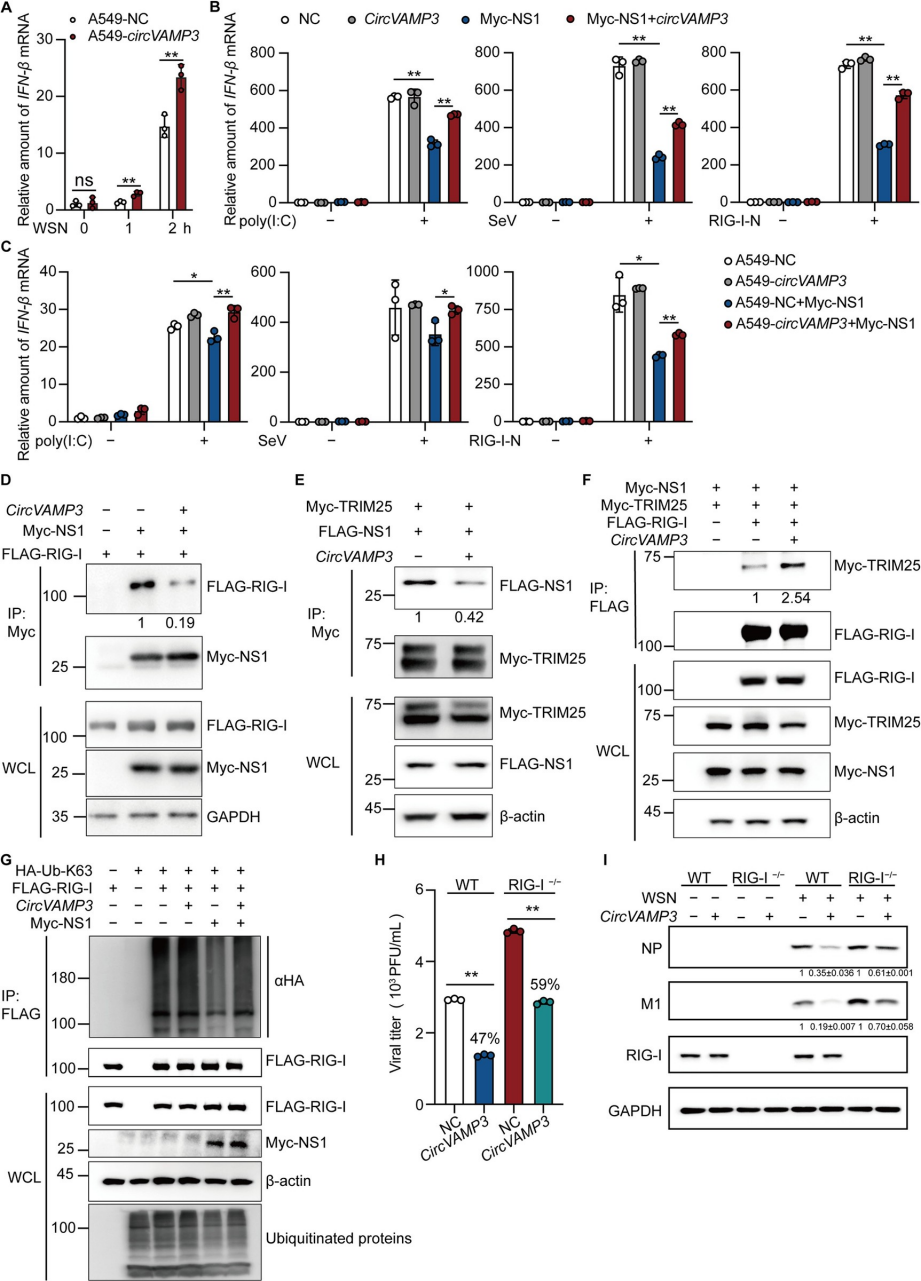
*CircVAMP3* restricts NS1 binding with RIG-I to restore IFN-β transcription. (A) A549 stable cell lines were grown in 12-well plates, followed by infecting with WSN at an MOI = 10, the cells were harvested at the indicated time points with Trizol for RT-qPCR to detect the IFN-β mRNA levels. (B and C) The cells were transfected with poly (I:C) (1 μg/mL) for 8 h (B and C, left panel), infected with SeV at 200 HAU/mL for 8 h (B and C, middle panel), or treated with IFN-β (B and C, right panel). Total RNA was prepared for RT-qPCR to detect the IFN-β mRNA levels. (D and E) Cell lysates were immunoprecipitated with Myc beads and immunoblotted with the indicated antibodies. (F and G) Cell lysates were immunoprecipitated with FLAG beads and immunoblotted with the indicated antibodies. (H and I) The 293T WT or RIG-I KO cells were grown in 24-well plates and transfected with *circVAMP3* expression plasmid or EV for 24 h. And then, infected with WSN at an MOI = 1 for 8 h. The gray analysis value in Fig I is the mean ± variance of three independent experiments. The data shown in A to C and H are presented as the means ± SD; *n =* 3; **p < 0.01. Data are representative of the results from at least three independent experiments.

NS1 plays an important role in inhibiting the RIG-I-mediated antiviral immune response [36,37]. We hypothesized that *circVAMP3* can abrogate the inhibitory effect of NS1 on the antiviral immune response. To test this hypothesis, we detected the expression of the IFN-β mRNA in A549-*circVAMP3* with or without WSN infection. The level of IFN-β mRNA was significantly increased in A549-*circVAMP3* cells compared with control cells, indicating that IAV-infected cells produced more IFN-β upon increasing *circVAMP3* (Fig 7A). In addition, 293T cells expressing pLC5-*circVAMP3* or control cells were transfected with poly (I: C) alone or together with the pcDNA-Myc-NS1. The RNA was prepared for RT-qPCR to detect the level of the IFN-β mRNA. We found that *circVAMP3* did not affect the IFN-β mRNA induced by poly(I:C). However, *circVAMP3* significantly enhanced the IFN-β mRNA expression, which was suppressed by NS1 protein (Fig 7B, left panel). Consistently, we observed that *circVAMP3* also antagonized the inhibitory effect of NS1 on the activation of IFN-β induced by SeV infection (Fig 7B, middle panel). To further determine the effect of *circVAMP3* on the RIG-I signaling pathway, 293T cells were transfected with RIG-I-N, a constitutively active form of RIG-I. Consistent with other stimuli, *circVAMP3* overexpression significantly rescued the decrease in IFN-β expression induced by NS1 protein (Fig 7B, right panel). Meanwhile, we also observed that *circVAMP3* overexpression antagonized the inhibitory effect of NS1 on the expression of IFN-β in A549-stable-overexpressing cells (Fig 7C).

Since we already identified that *circVAMP3* interacts with NS1 1–84 aa region (RBD domain) and NS1 RBD domain can interact with RIG-I and TRIM25[36,38]. Next, we sought to determine whether *circVAMP3* affected the association of NS1 with RIG-I or TRIM25. Firstly, FLAG-RIG-I and Myc-NS1 were co-expressed with or without pLC5-*circVAMP3*, and cell lysates were subjected to a coimmunoprecipitation assay. We found that *circVAMP3* reduced the association of Myc-NS1 with FLAG-RIG-I (Fig 7D). Meanwhile, FLAG-NS1 and Myc-TRIM25 were co-expressed with or without *circVAMP3*, and cell lysates were subjected to a coimmunoprecipitation assay. We found that *circVAMP3* reduced the association of Myc-TIRM25 with FLAG-NS1 (Fig 7E). And then, based on previous studies reported that NS1 specifically inhibits TRIM25-mediated RIG-I CARD ubiquitination, hence suppressing RIG-I signal transduction [38,39]. Therefore, we investigated whether *circVAMP3* affected the inhibition of NS1 on the association of RIG-I with TRIM25. We transfected Myc-NS1, Myc-TRIM25, FLAG-RIG-I, with or without pLC5-*circVAMP3*, and cell lysates were subjected to a coimmunoprecipitation assay. We found that *circVAMP3* expression impaired the inhibition of Myc-NS1 on the association of FLAG-RIG-I with Myc-TRIM25 (Fig 7F). Finally, we observed K63-linked RIG-I ubiquitination to observe RIG-I activation. As shown in Fig 7G, *circVAMP3* restored RIG-I K63 ubiquitination that was suppressed by NS1. These data confirmed that *circVAMP3* rescues RIG-I activation by diminishing the interaction of NS1 with RIG-I. To further clarify if *circVAMP3* can play an antiviral role through the RIG-I signaling pathway, RIG-I-depleted 293T cells were used to detect the effect of *circVAMP3* upon IAV infection. As shown in Fig 7H and 7I, in 293T cells without RIG-I depletion, overexpression of *circVAMP3* significantly inhibited viral titers and IAV protein levels, consistent with previous findings. However, the RIG-I deficient 293T cells had significantly higher titers and protein levels than wild-type cells. Although *circVAMP3* overexpression still significantly inhibited the proliferation of influenza virus, the inhibition rate was reduced in RIG-I-deficient 293T cells.

**Fig 8:**
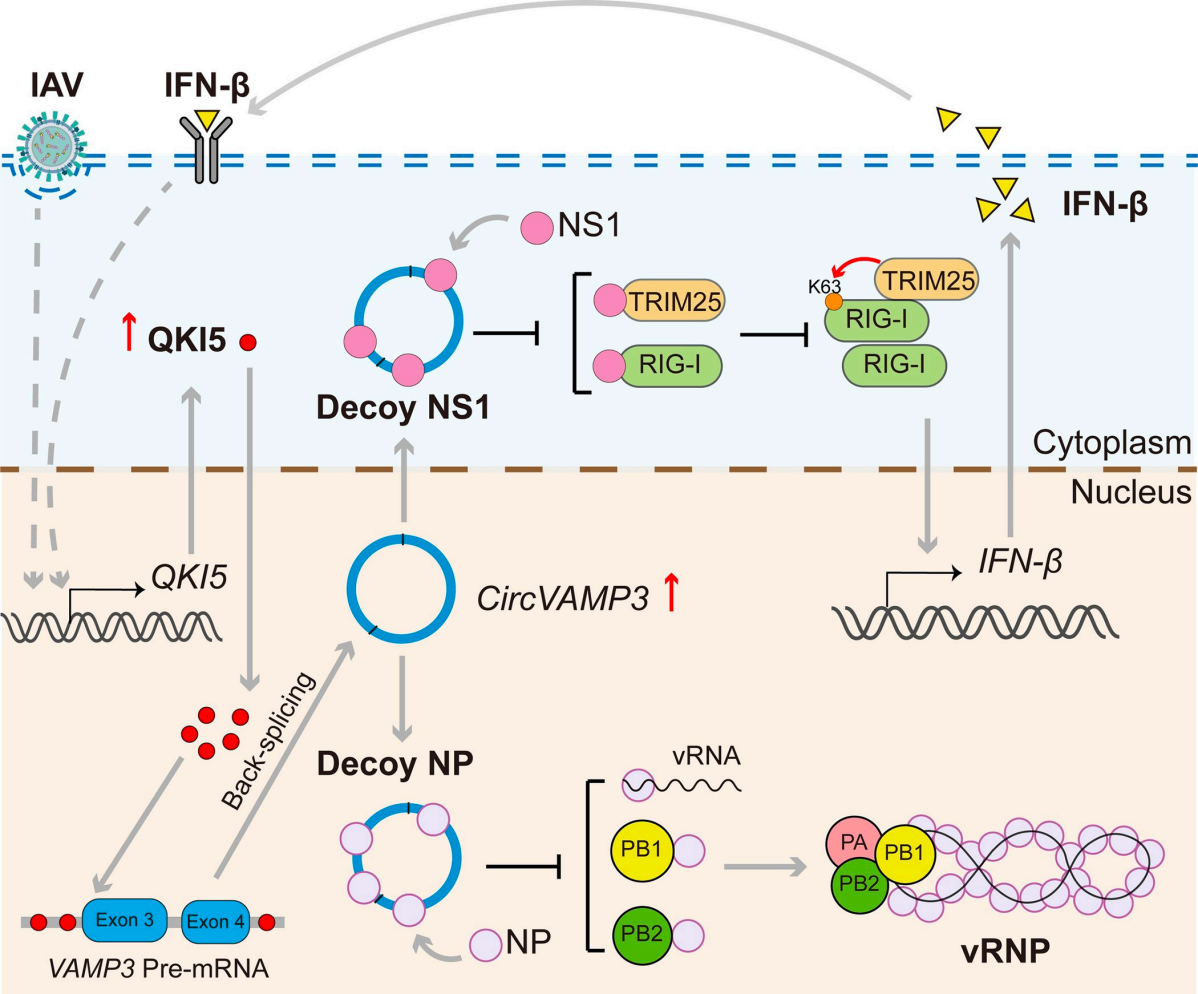
Hypothesized model by which circVAMP3 contributes to inhibiting IAV replication. IAV infection and IFN-β stimulation triggers the transcription of *QKI-5* upregulated. The upregulated QKI-5 mediates *circVAMP3* biogenesis. *CircVAMP3* inhibits vRNP activity by disturbing the interaction of NP with PB1, PB2, or vRNA and restores the activation of IFN-β by alleviating the inhibitory effect of NS1 on the RIG-I signaling pathway.

In conclusion, we identified that a circular RNA, *circVAMP3*, is upregulated by IAV infection or IFN-β treatment. Additionally, IAV- or IFN-β-mediated upregulation of QKI-5 mediates *circVAMP3* biogenesis. Functional *in vitro* and *in vivo* analyses indicate that *circVAMP3* plays a vital role in restricting IAV replication. Mechanistically, we first discovered that *circVAMP3* inhibits vRNP activity by disturbing the interaction of NP with PB1, PB2, or vRNA and restores the activation of IFN-β by alleviating the inhibitory effect of NS1 on the RIG-I signaling pathway (Fig 8).

## Discussion

In this report, we reveal a novel circRNA-mediated antiviral mechanism in which *circVAMP3* directly inhibits viral replication by acting as a decoy to IAV NP and NS1 proteins. We present evidence that *circVAMP3* inhibits the interaction of NP with PB1, PB2, or vRNA to impair vRNP activity, as well as antagonize NS1-mediated suppression of RIG-I activation to restore antiviral innate immunity.

*CircVAMP3* plays multiple functions in different biological processes. *CircVAMP3* is highly expressed in alveolar rhabdomyosarcoma cells and potentially regulates cell cycle progression through AKT-related pathways [40]. Conversely, *circVAMP3* is significantly downregulated in hepatocellular carcinoma tissues, which inhibiting proliferation by facilitating stress granule formation [41]. However, the well-known function of *circVAMP3* is still limited to neoplasm regulation. In this study, we unveil a novel molecular mechanism of *circVAMP3* during viral infection. We reported that *circVAMP3* can regulate the replication of IAV. Contrary to the widely reported role of circRNAs as miRNAs sponges to indirectly regulated gene expression, we did not observe the upregulated VAMP3 protein levels upon *circVAMP3* overexpressed. Consistently, VAMP3 mRNA remained unaffected in both *cricVAMP3* knocking-down or overexpression cell lines [42], suggesting that *circVAMP3* does not act as a miRNA sponge.

The mechanisms of circRNAs generation, stability, and degradation remain uncleared. QKI belongs to the signal transduction and activation of RNA (STAR) family of KH domain-containing RBP with three major isoforms: QKI-5, QKI-6, and QKI-7[43]. In our study, we observed that overexpressing QKI-5 enhances *circVAMP3* generation, while *QKI* knockdown suppressed *circVAMP3* generation. It’s consistent with the result of the *QKI* knockdown transcriptome [43]. Additionally, IAV infection and IFN-β stimulation upregulate QKI-5 to promote *circVAMP3* biogenesis. Since IAV infection induces dose-dependent upregulated of IFN-β gene expression [44], IAV infection may promote *circVAMP3* generation by inducing IFN-β expression. Notably, QKI is frequently downregulated in lung cancer and associated with a poorer prognosis [30]. Thus, QKI-5 may also facilitate *circVAMP3* formation in cancer-related processes. Moreover, it is unclear whether IFN-β facilitates *VAMP3* pre-mRNA expression, suggesting *circVAMP3* may serve as an ISG product or another mechanism promoting its generation. What’s more, we observed that linear VAMP3 mRNA and *circVAMP3* had longer half-lives than RIG-I mRNA. It has been reported that VAMP3 mRNA contains m6A modifications, which may stabilize its stability. However, *circVAMP3* is insensitive to m6A-mediated regulation [40]. Previous studies have shown a rapid decrease in circRNAs abundance after poly (I:C) treatment or virus infection [45]. However, there are conflicting reports on the regulation of circRNAs levels during these treatments [46]. Similarly, we found both upregulation and downregulation of circRNAs during IAV infection. The underlying mechanisms controlling the levels and half-life of specific circRNAs need further investigated.

NcRNAs, including lncRNAs [19,47–49] and small ncRNAs [50,51], are induced by IFN and play roles in antiviral immunity [52]. For instance, lncBST2 promotes the expression of antiviral ISG BST2, inhibiting virion release [47,53]. Our previous study showed that lnc-ISG20 acts as a miR-326 sponge to increase ISG20 protein expression, inhibiting IAV replication [19,54]. While lnc-MxA interferes with the binding of IRF3 and p65 to the IFN-β promoter, negatively regulating the antiviral immune response [49]. Interestingly, miR-122 inhibits hepatitis C virus (HCV) replication but is suppressed by IFN [50,55]. We found that *circVAMP3* is upregulated by IFN-β. Previous studies have reported upregulation of VAMP3 by LPS and IFN-γ priming [56], while its paralog gene VAMP5 is also an IFN-γ-stimulated gene [57]. Together, these findings suggest that IFN-induced ncRNAs, including circRNAs, participate in the antiviral process as ISGs, complementing the traditional definition of ISGs.

Like other ncRNAs, circRNAs can work as RNA decoys for proteins. The biological function and detailed mechanisms of circRNAs remain unclear. Bioinformatics analysis has been used to predict the potential functions of circRNAs, but it has shown that circRNAs do not tend to function as miRNA sponges or bind RBPs like linear mRNAs [58,59]. Nevertheless, interactions between circRNAs and proteins or miRNAs have been reported in various studies [23]. Our study also revealed that *circVAMP3* can directly interact with viral proteins. Recent studies have focused on how circRNAs binding to host proteins indirectly affects the response to viral infection [60]. We were the first to report that circRNA can act as an RNA decoy by directly interacting with viral proteins to inhibit their function. The concept of using decoy ncRNAs to sequester proteins originated from bacteria with the discovery of small RNAs acting as decoys [61]. Long ncRNAs have also been identified as decoy molecules that bind and sequester proteins, inhibiting the proteins’ normal functions [62,63]. As a new member of long ncRNA, circRNAs have been reported to act as RNA decoys for host proteins [64]. In our study, we found that *circVAMP3* acts as a decoy for viral proteins NP and NS1. Our results demonstrated that ncRNAs act as RNA decoys not only for host proteins but also for viral proteins.

The structure of ncRNAs plays a crucial role in their interaction with proteins. RNA typically interacts with protein through base stacking, hydrogen bonding, electrostatic interactions, and hydrophobic interactions [65]. The ncRNAs were thought to predominantly adopt secondary or tertiary structures, suggesting that the primary sequence of RNA is critical for protein binding, while the secondary structure enhances the affinity of RNA-protein interaction [61]. Due to the unique tertiary structure of circRNAs, their ability to bind proteins may be more complex than previously investigated. In our study, we found that *circVAMP3* preferentially binds to the C-terminal region of NP rather than the RNA-binding domain, interrupting NP binding with vRNA. It would be worthwhile to investigate in future studies which portion of the unique tertiary structure of *circVAMP3* determines its interaction with NP. These findings indicate that traditional nucleotide sequencing-based methods used to analyze linear mRNAs binding to protein may not be suitable for studying circRNA-protein interactions. Furthermore, our study demonstrated that *circVAMP3* antagonized the association of NP with PB1 and PB2, but not NP self-polymerization. This is consistent with previous reports, as RNA (vRNA and 24-nucleotide ssRNA) binding itself does not affect NP self-polymerization [5,66]. Interestingly, although all four segments of *circVAMP3* significantly bind to NP or NS1 protein, none of them significantly inhibit IAV replication compared to *circVAMP3* itself (S7 Fig). This suggests that the primary structure of *circVAMP3* may support strong binding, while the tertiary structure may play a role in its inhibition function. However, the dynamic tertiary structure of circRNAs can be influenced by various factors in the cellular environment, leading to variations in different cell lines, tissues, and developmental stages [67]. Studying circRNAs in vitro may cause changes in their tertiary structures, making it challenging to reveal their true dynamic interactions in situ. Therefore, more research is needed to understand how circRNAs bind to different proteins.

CircRNAs hold promise as a new type of molecular drug, but further research is needed. *CircVAMP3*, as a stable antiviral factor with multiple binding sites for viral proteins, has demonstrated suppression of various IAV strains by inhibiting viral NP and NS1 protein functions. Further evaluation of *circVAMP3* potential as an antiviral drug is warranted. CircRNAs have attracted significant attention due to their involvement in various biological processes. However, many circRNAs functions during viral infection remain unknown [68,69], and further investigations are needed to understand their interaction with host immune system in the antiviral defense response. It is also important to consider the spatiotemporal regulation of circRNA function, as some circRNAs may work by changing localization patterns or over time. Exploring the physiological function of circRNAs will enhance our understanding and provide potential therapeutic strategies for influenza virus infection.

## Materials and methods

### Mice and ethics statement

Female BALB/c mice (4–6 weeks old) were purchased from Beijing Vital River Animal Technology Co., Ltd. Mice were maintained under specific pathogen-free (SPF) conditions. Animal studies were approved by the Committee on the Ethics of Animal Experiments of the Institute of Microbiology, Chinese Academy of Sciences (IMCAS) and conducted in compliance with the recommendations in the Guide for the Care and Use of Laboratory Animals by the IMCAS Ethics Committee (permit number PZIMCAS2019001). The animals were housed and bred in SPF mouse facilities in compliance with ethical guidelines, and all animal experiments were approved by the Experimental Animal Ethics and Welfare Committee of IMCAS.

### Cell lines and virus strains

Madin-Darby canine kidney cells (MDCK) (ATCC CCL-34), human lung adenocarcinoma epithelial cells (A549) (ATCC CCL-185), human embryonic kidney HEK293T cells (293T) (ATCC CRL-3216), and Lewis lung carcinoma LL/2 (LLC1) cells (ATCC CRL-1642) of C57BL were purchased. The 293T-IAV-Luc cell line is constructed by our previous study [70]. The cells were maintained in Dulbecco’s modified Eagle’s medium (DMEM, Gibco) supplemented with 10% (v/v) heat-inactivated fetal bovine serum (FBS, Lonsera, 711-001S, www.lonsera.cn), 100 U/mL penicillin, and 100 ug/mL streptomycin at 37°C in an atmosphere containing 5% CO_2_. The influenza A/WSN/1933 (WSN), influenza A/California/2009/04 (CA04), and influenza A/Puerto Rico/8/1934 (PR8) strains were propagated in MDCK cells. A/Darwin/6/2021/H3N2, A/chicken/Hebei/LC/2008(H9N2) (HB08) strain and Sendai virus (SeV) was propagated in 9-day-old SPF chicken embryonated eggs purchased from Beijing Vital River Animal Technology Co., Ltd. (licensed from Charles River) at 37°C for 72 h.

### Antibodies and reagents

The rabbit anti-NP polyclonal antibody and mouse anti-M1 monoclonal antibody were generated as previously described [71]. The rabbit anti-NS1 polyclonal antibody was generated as previously described [72]. The mouse anti-FLAG M2 monoclonal antibody (Sigma-Aldrich, USA), mouse anti-Myc monoclonal antibody (Santa Cruz Biotechnology, USA), mouse anti-His monoclonal antibody (Beyotime, China), rabbit anti-IAV PB1 polyclonal antibody (GeneTex, USA), rabbit anti-IAV PB2 polyclonal antibody (GeneTex, USA), mouse anti-Lamin B1 monoclonal antibody (Santa Cruz Biotechnology, USA), mouse anti-β-actin monoclonal antibody (TransGen Biotech, China), mouse anti-GAPDH monoclonal antibody (Abcam, UK), HRP-conjugated goat anti-mouse IgG (Beyotime, China), HRP-conjugated goat anti-rabbit IgG (Beyotime, China), HRP-conjugated rabbit anti-mouse IgG (Cell Signaling Technology, USA), and HRP-conjugated mouse anti-rabbit IgG (Cell Signaling Technology, USA) were purchased from the indicated companies. Poly(I:C) sodium salt (Sigma, USA), human IFN-β (PeproTech, USA), RNase R (Lucigen, USA), RiboLock RNase Inhibitor (Thermo Fisher Scientific, USA), protease inhibitor cocktail (Roche, Switzerland), isopropyl-β-D-thiogalactopyranoside (IPTG; Calbiochem, USA), and tolylsulfonyl phenylalanyl chloromethyl ketone (TPCK)-trypsin (Sigma-Aldrich, USA) were purchased from the indicated companies.

### Plasmids

The *circVAMP3* sequence was cloned into the lentiviral pLC5-ciR-GFP vector (Gene-seed, China) to construct an overexpression plasmid. The full-length sequences of QKI-5 and EIF4A3 were cloned into pcDNA3.1-Myc (+) and pcDNA3.1-FLAG (+), respectively. The full-length sequences of NP and NS1 from A/WSN were cloned into pET-21a-His (+). The full-length sequence of M1 from A/WSN was cloned into pET30a-His. Truncated NP sequences (aa 1–181 and 182–498) were cloned into pcDNA3.1-FLAG (+). Truncated NS1 sequences (aa 1–83 and 84–230) were cloned into pcDNA3.1-Myc (+). The *td* 3’intron-*circVAMP3*-*td* 5’ intron sequence was cloned into pBluescript II KS (+) for *in vitro* transcription. The *circVAMP3* sequence was cloned into pHBAAV-CMV-circRNA-EF1-ZsGreen by HANBIO Co., Ltd. to package AAV6-*circVAMP3*. The full-length sequences of PB1 and PB2 from A/WSN were cloned into pcDNA3-FLAG, and NP was cloned into pcDNA3-FLAG and pCMV-Myc [73]. The full-length sequence of NS1 from A/WSN was cloned into pCMV-Myc and pcDNA-FLAG, and the resulting plasmids were named pCMV-Myc-NS1 and pcDNA-FLAG-NS1, respectively. RIG-I was cloned into pcDNA-FLAG, and the resulting plasmid was named pcDNA-FLAG-RIG-I. The NS cRNA promoter and luciferase were cloned into the pHH21 vector to produce the pHH21-vNS-Luc plasmid [74]. pFLAG-CMV2-RIG-I-N, pRL-TK (Renilla), Luc-IFN-β, pSIH1-H1-GFP, pCMV-VSVG, pRSV-REV, and pMDIG were stored in the laboratory. The plasmids used for the A/WSN vRNP activity assay (pcDNA-PB2, pcDNA-PB1, pcDNA-PA, and pCAGGS-NP) and the WSN package plasmids were gifts from Yoshihiro Kawaoka (University of Wisconsin).

### RNA sequencing analysis

A549 cells were infected with WSN, CA04, or PR8 at an MOI of 1 or incubated with PBS (mock-infected) for 8 h. Total RNA was extracted with TRIzol reagent (Thermo Fisher Scientific, USA). The purity of the RNA samples was evaluated using agarose gel electrophoresis and a NanoDrop One spectrophotometer (Thermo Fisher Scientific, USA). RNA with an RNA Integrity Number (RIN) > 7, as assessed with an Agilent 2200 bioanalyzer (Agilent Technologies, USA), was screened. The rRNA was removed with a Ribo-Zero rRNA Depletion Kit (RiboBio, China), and linear RNA was removed by RNase R treatment. In addition, 1 μg of purified RNA was used for circRNA library construction. After removing reads that contained adapter sequences and low-quality reads, circRNAs were identified from the remaining reads using CIRI2 software. The remaining junction reads were aligned to human circRNAs in the circBase database (http://www.circbase.org/). Data from both the IAV- and mock-infected cells were used to identify differentially expressed circRNAs based on back splicing junction reads per million mapped reads (RPM) with a fold-change > 2 and *p*-value < 0.05. All raw data from these RNA-seq data were deposited in the National Center for Biotechnology Information (NCBI) Short Read Archive (SRA) database with accession number PRJNA750806.

### RT-PCR and RT-qPCR

Total RNA was isolated from cells with TRIzol reagent, treated with or without RNase R (1 U/μg), and subjected to RT-qPCR to detect *circVAMP3*. First-strand cDNA was synthesized with Hifair II 1st Strand cDNA Synthesis SuperMix for qPCR (Yeasen, China). RT-qPCR was performed with Hieff qPCR SYBR Green Master Mix (Yeasen, China) according to the manufacturer’s protocol. Relative gene expression was quantified with GAPDH as the internal control to normalize.

### Nuclear and cytoplasmic fractionation

Cytoplasmic and nuclear fractionation was performed with nuclear and cytoplasmic extraction reagents (Applygen, China) according to the manufacturer’s instructions. Briefly, A549 cells were lysed in CEB-A buffer on ice for 15 min. After centrifugation at 2,000 × g for 10 min, the supernatant was collected as the cytoplasmic extract. The pellet was suspended in PBS on ice and washed three times. After centrifugation at 2,000 × g for 10 min, the pellet was suspended in CEB-B buffer on ice, incubated for 30 min, and subsequently centrifuged at 12,000 × g for 5 min, after which the supernatant was carefully collected as the nuclear extract.

### RNA fluorescence *in situ* hybridization (FISH)

A biotin-labeled oligonucleotide probe for *circVAMP3* was synthesized by Tsingke Co., Ltd. (China). Cultured cells on slides were washed in PBS and fixed in 4% polyformaldehyde at room temperature for 30 min. The fixed cells were then treated with 0.5% Triton X-100 and then incubated with 50% formamide/2×SSC for 20 min. Hybridization was performed at 42°C for 16 h with the biotin-labeled *circVAMP3* probe. After washing with 50% formamide/2×SSC, the slides were hybridized with FITC-conjugated streptavidin (Bioss, China) at 37°C for 2 h in a dark chamber and washed. Then, the slides were incubated with 4% BSA, stained with DAPI (Thermo Fisher Scientific, USA), and sealed with Vectashield (Vector Laboratories, USA). Images were acquired using fluorescence microscopy (Leica SP8 confocal microscopy, Leica, Germany).

### Transfection

Cells were plated and transfected with the indicated plasmid using Lipofectamine 2000 Transfection Reagent (Thermo Fisher Scientific, USA) for 24 h according to the manufacturer’s instructions. A549 cells were plated and transfected with 50 nM of the indicated siRNA using RNAiMax (Invitrogen, USA).

### Plaque assay

MDCK cells were seeded in 12-well plates, grown in monolayers, and infected with the indicated virus in serum-free DMEM. After adsorption, the cells were washed twice with PBS and overlaid with MEM containing 2 μg/μL TPCK-treated trypsin and 1% low-melting-point agarose at 4°C for 10 min, after which they were incubated at 37°C with 5% CO_2_ for 72 h. Then, the cells were fixed with a 4% formaldehyde solution overnight at RT and stained with a 0.05% (*w/v*) crystal violet solution. Viral plaques were counted, and viral titers were calculated.

### Generation of stable overexpression and knockdown cell lines

Stable overexpression and knockdown cell lines were generated by transfecting 293T cells with the lentiviral packaging vector (pRSV, pVSVG, and pMDIG) and plc-ciR-*circVAMP3* or pSIH-*circVAMP3*-shRNA for 48 h. Then, the virus-containing supernatant was collected and used to infect A549 cells for 72 h.

### RNA synthesis and purification

QKI, EIF4A3, or DHX9 siRNAs and truncated fragments of biotinylated *circVAMP3* were synthesized by Sangon Biotech (China). *CircVAMP3* was generated *in vitro* using the permuted intron-exon (PIE) method, and the sequence was designed to contain autocatalytic splicing *td* introns [75]. *CircVAMP3* was synthesized with a MEGAscript T7 transcription kit (Ambion, USA) at 37°C for 12 h. Biotin-labeled *circVAMP3* was synthesized, and dNTP was replaced with Biotin RNA Labeling Mix (Roche, Switzerland). Transcribed *circVAMP3* and biotin-*circVAMP3* were purified with RNeasy Mini columns (Qiagen, Germany) and then treated with RNase R at 37°C for 2 h to degrade linear RNA byproducts. After purification with RNeasy Mini columns, RNA quality was assessed on a 5% acrylamide/8 M urea gel. The goal band was extracted from the gel with a Zymoclean Gel Recovery Kit (Zymo Research, USA).

### Protein expression and purification

For the purification of His-NP, His-NS1, and His-M1 proteins, the pET-21a-His-NP, pET-21a-His-NS1, and pET30a-His-M1 plasmids were transformed into *E*. *coli* Rosetta (DE3) cells, respectively. Transformants were cultured at 37°C on LB medium containing 50 μg/mL kanamycin for 14 h. Single colonies were picked, inoculated into LB (K^+^) medium, grown to an optical density at 600 nm of 0.6–0.8, and then protein expression was induced with 0.2 mM IPTG at 16°C for 24 h. Harvested cells were lysed by sonication, and then the proteins were purified as previously described [76]. Purified proteins were detected using SDS-PAGE.

### Immunoblotting and immunoprecipitation

The cells were lysed with ice-cold lysis buffer (20 mmol/L HEPES, pH = 7.4, 150 mmol/L NaCl, 10% glycerin, 1% Triton X-100, and 1 mmol/L EDTA) supplemented with a protease inhibitor cocktail and RNase inhibitor. Protein separation was conducted by 10% SDS-PAGE, and the proteins were transferred onto a PVDF membrane that was then immunoblotted with the indicated antibodies. The blots were visualized with ECL substrate (Tanon, USA). ImageJ software (National Institutes of Health, USA) was used to perform the gradation analysis. For immunoprecipitation, the cells were lysed with lysis buffer supplemented with protease inhibitor cocktail and RiboLock RNase inhibitor on ice for 30 min. The lysates were immunoprecipitated with the indicated antibodies and Protein A-Sepharose (Sigma-Aldrich, USA) or Ni-NTA beads (TransStart, China) and anti-c-Myc agarose affinity gel (Myc beads) or anti-FLAG M2 affinity gel (FLAG beads) for 5 h. The beads were rinsed five times with ice-cold wash buffer (20 mmol/L HEPES, pH 7.4, 500 mmol/L NaCl, 10% glycerin, 1% Triton X-100, and 1 mmol/L EDTA), the bound RNA was eluted and subjected to RT-qPCR and normalized with 1% input the indicated RNA, and the proteins were detected using immunoblotting.

### Virus attachment and internalization assay

The cells in 12-well plates were washed with PBS (pH = 7.2) three times and then were infected with WSN virus (MOI = 5 or 10) on ice at 4°C for 1 h. After washing with ice-cold PBS (pH = 7.2) three times to remove unbound viruses, the cells were frozen and thawed three times to release the bound viruses of the cells’ surface into the supernatant. Using 1 mL DMEM collected the viruses and then titrated by a plaque assay on MDCK cells. Or after the second washing, the cells were harvested with lysis buffer for immunoblotting assay with anti-M1 mAb. Both assays help to detect the virus attachment. Or after the first washing, the cells were infected with WSN (MOI = 10) and cultured at 37°C for 15, 30, and 60 min to allow for internalization. The cells were then washed with ice-cold PBS-HCl (pH = 1.3) five times to remove the attached but not-yet-internalized virions. And then, subjected to immunoblotting with anti-M1 mAb.

### RNA pulldown assay

A biotin-labeled *circVAMP3* probe targeting the unique *circVAMP3* back splicing junction (BSJ) and control probe were purchased from Sangon Biotech (Shanghai, China). They were incubated with streptavidin C1 magnetic beads (Invitrogen, USA) for 15 min at room temperature and then incubated with cell lysates or *in vitro* transcribed *circVAMP3* and purified NP or NS1 protein at 4°C for 4 h. The bound proteins were then detected by immunoblotting.

### Luciferase reporter assay

The 293T cells were seeded in 24-well plates and co-transfected with pcDNA-PB2, pcDNA-PB1, pcDNA-PA, pCAGGS-NP, pHH21-vNS-Luc, and pRL-TK(Renilla) with pLC5-*circVAMP3* or pLC5-ciR-GFP for 30 h. The cells were collected and subjected to a luciferase assay with a Dual-Luciferase Assay Kit (Promega, USA). Relative luciferase activity was calculated using Renilla luciferase as an internal control.

### Silver staining and mass spectrometry

The RNA pulldown samples were separated by 10% SDS-PAGE, after which the gels were stained with a Pierce Silver Stain for Mass Spectrometry Kit (Thermo Fisher Scientific, USA). The densest bands were subjected to analysis using nanoscale liquid chromatography-tandem mass spectrometry (nano-LC-MS/MS, LCQ Deca XP Plus; Thermo, USA). The data were analyzed by performing a MASCOT search against the SWISS-PROT human database and IAV database.

### Animal modeling and *in vivo* infection

The mice were anesthetized with tribromoethanol, after which AAV6 expressing *circVAMP3* purchased from Hanheng Company (Hanheng Biotechnology, China) was intratracheally instilled into the mice (5×10^10^ vg/mL per mouse). After 21 days, the mice were challenged with the WSN strain at 10^4^ PFU per mouse by nasal drip. Body weights and survival were recorded up to 14 days post-infection (d p.i.). The dry and wet lung weights of the mice were recorded on days 0, 3, 5, and 7. The lungs of the mice were harvested and placed in 10% buffered formalin for histological sectioning or 1 mL of PBS for detection of the viral titer. The fixed samples were then embedded in paraffin and used for immunohistochemistry (IHC) with the anti-M1 mAb or hematoxylin and eosin (H&E) staining. And then, the pathological scores of H&E were evaluated in a blinded manner by five graders who were unaware of the specific group under review. The sections were evaluated based on five indices, including pulmonary interstitial edema, alveolar edema, inflammatory cell invasion, alveolar hemorrhage areas, and hyaline membrane development, and the score of each index ranged from 0 to 3 points.

### Quantification and statistical analysis

Statistical analyses were performed using Prism version 8.0 software (GraphPad Software, USA). The statistical significance of differences between groups was determined using unpaired Student’s *t*-test. Data are presented as the means ± SD. ns, not significant. A significant difference was determined when *p < 0.05 or **p < 0.01.

## Supporting information

S1 Fig CircRNA profiles obtained after IAV infection and IFN-β administration reveal the upregulation of *circVAMP3*. (A, B) A549 cells were infected with IAV (WSN, CA04, or PR8) at an MOI of 1 for 8 h. Total RNA was treated with RNase R and analyzed by RNA sequencing. (A) Volcano plots illustrating differentially expressed circRNAs in WSN-, CA04-, or A/PR8-infected cells compared to mock-infected cells. (B) Venn diagram showing the distribution of differentially expressed circRNAs in IAV-infected cells. The numbers inside the points of intersection indicate the number of common or specific circRNAs. (C, D, E) 293T cells were infected with SeV at 200 HAU/mL for 8 h (*n =* 3) (C), transfected with poly (I:C) (0.5 μg/mL or 2 μg/mL) for 8 h (*n =* 3) (D), or treated with IFN-β (10 ng/mL or 40 ng/mL) for 12 h (*n* = 3) (E), after which the total RNA was extracted, treated with or without RNase R, and quantified using RT-qPCR to detect *circVAMP3*. Data shown in panels C-E were normalized to GAPDH. Data are presented as the means ± SD. **p < 0.01.
(TIF)

S2 Fig IAV increases the translation and transcription of QKI-5. (A) Trans-acting factors, namely, RBPs, promote circRNA biogenesis. (B, C, D) A549 cells were infected with or without WSN at an MOI of 1. (A) The cell lysates were harvested at 2 or 4 h and subjected to quantitative proteomics analysis. Proteins associated with circRNA biogenesis are listed in order of fold change (FC). (B) Schematic illustration of putative binding sites for the three RNA-binding proteins (QKI-5, EIF4A3, and DHX9) in *circVAMP3* flanking introns. (C) The cell lysates were collected and subjected to immunoblotting with the indicated antibodies. (D) Total RNA was extracted, and QKI-5, DHX9, and EIF4A3 were quantified using RT-qPCR. (E) siRNA-mediated knockdown of *circVAMP3* flanking intron-binding proteins with two independent siRNAs, respectively. Western blots confirmed the knockdown of QKI-5 (E, upper left panel), EIF4A3 (E, upper middle panel), and DHX9 (E, upper right panel). The knockdown efficiency of QKI-5 (E, lower left panel), EIF4A3 (E, lower middle panel), and DHX9 (E, lower right panel) was detected using RT-qPCR. (F) Western blot of overexpressed QKI-5 and EIF4A3. Data are presented as the means ± SD. **p < 0.01.
(TIF)

S3 Fig Transient overexpression of *circVAMP3* inhibited IAV replication. (A) The 293T cells were grown in 6-well plates and transfected with 4 μg *circVAMP3* plasmid for 24 h. And then, infected with WSN (MOI = 0.5) for 8 h. The cell supernatants were collected for plaque assays (B), and cell lysates were used for immunoblotting assays (C). (D) The cyclization site of the redesigned *circVAMP3*. (E) Verification of the product of the *circVAMP3* overexpression construct. Original construct, third and fourth exon cyclization; new construct, original junction shifted 15 nt to the third exon. (F, upper panel) Semi-quantification of stably overexpressing cell lines constructed by packaging lentivirus with a new *circVAMP3* overexpression vector using reverse primer amplification products separated by agarose gel electrophoresis. (F, lower panel) Sequencing results of single strip cutting in Figure F. (G) The 293T cells in 12-well plates were transfected with a gradient plasmid quantity as indicated in the figure. After 24 h, cells were infected with WSN (MOI = 0.5) for 8 h. The data shown in A and B are presented as the means ± SD; *n =* 3; **p < 0.01. Data are representative of the results from at least three independent experiments.
(TIF)

S4 Fig Transient overexpression of *circVAMP3* inhibited IAV replication. (A) The LLC1 cells were infected with WSN (MOI = 1) for 12 h, followed by RT-qPCR to detect the level of the indicated RNA, using the Vamp3 mRNA and *circIpo11* as positive controls. (B) 293T cells and lungs of BALB/c mice were not infected. (C) LLC1 cells were infected with WSN (MOI = 5) or treated with PBS for 4 h. (D) Using lentivirus to infect LLC1 cells. RT-qPCR confirmed that LLC1 cells stably overexpressed *circVAMP3*. (E and F) The LLC1 cells, stably overexpressed *circVAMP3*, were infected with WSN (MOI = 0.5) for 12 h and 24 h. The supernatants were collected for plaque assay (E) and the cell lysates were harvested for immunoblotting (F). Data shown in E is presented as the mean ± SD. *n =* 3; **p < 0.01. These data represent the results of at least three independent experiments.
(TIF)

S5 Fig Mass spectrometry analysis of the *circVAMP3*-binding proteins. (A, B) A549 cells were infected with WSN. Cell lysates were subjected to a pulldown assay with biotin-labeled *circVAMP3* or unlabeled *circVAMP3* as the control. The bound proteins were visualized using silver staining. The arrows indicate the bands that were subjected to mass spectrometry analysis. The *circVAMP3*-bound human proteins (A) and IAV-H1N1 proteins (B) are listed in order of score and expected value. *CircVAMP3* was predicted to interact with NP (C) and NS1 (D) by the HADDOCK 2.4 website. The *circVAMP3* secondary structure was predicted according to the minimum free energy (MFE). Red indicates strong confidence in the prediction. The 3D structures of NP and NS1 were derived from the I-TASSER server by homology modeling.
(TIF)

S6 Fig *CircVAMP3* inhibits IAV replication in the early stages of viral infection. (A) A549 cells stably overexpressing pLC5-*circVAMP3* or controls, WSN (MOI = 1) infection and cell lysates were collected at 4 hp.i. for RT-qPCR to detect the level of viral M1 and NP RNAs. (B) The 293T cells were transfected with pLC5-*circVAMP3* or pLC5-ciR-GFP for 24 h, followed by being infected with WSN (MOI = 1), and cell lysates were collected at 2 hp.i. and 4 hp.i. for RT-qPCR to detect the level of viral M1 RNAs. (C) Transfected the pLC5-*circVAMP3* or pLC5-ciR-GFP into 293T cells at 12-well plates. And then, infected with WSN (MOI = 5), and cell lysate was harvested at the indicated time points. One-tenth of the cell lysate was taken for total protein level detection, and the rest was separated according to the operation instructions. And then, immunoblotted with the indicated antibodies. (D to F) The A549 stable cell lines were infected with WSN on the ice at 4°C for 1 h, followed by a neutral wash (ice-cold PBS = 7.2). E and F MOI = 10. The cells were harvested for plaque assay (D) or immunoblotting (E). (F) After a neutral wash, the cells were cultured with DMEM at 37°C for the indicated time points, followed by an acidic wash (ice-cold PBS-HCl = 1.3) before cell lysis. The internalized WSN was detected by immunoblotting. The data shown in A, B, and D are presented as the means ± SD; *n =* 3; ns is no significant difference, *p < 0.05, **p < 0.01.
(TIF)

S7 Fig The single segment of *circVAMP3* can’t inhibit IAV replication. (A and B) The 293T cell were spread into 12-well plates for 16 h, and then transfected with pLC5-*circVAMP3* or pLC5-ciR-GFP for 16 h. The rest of the cells were transfected with indicated 100 pmoL RNA segments for 8 h, respectively. Finally, all cells were infected with WSN at an MOI of 0.5 for 12 h. (A) The supernatants of infected cells were collected for plaque tests to determine the virus titer. (B) Cell lysates were collected and used for immunoblotting with corresponding antibodies.
(TIF)

S1 Table Excel spreadsheet containing, in separate sheets, the underlying numerical data and statistical analysis for Figs panels 1B, 1C, 1F, 1G, 1H, 2C, 2D, 2E, 2F, 3A, 3B, 3D, 3E, 3G, 3H, 3J, 3L, 4C, 4D, 4F, 4G, 4J, 5C, 5D, 5E, 5F, 5G, 5J, 5K, 5L, 5M, 6A, 6B, 6C, 6G, 7A, 7B, 7C, 7H, S1C, S1D, S1E, S2D, S2E, S3A, S3B, S4C, S4E, S6A, S6B, S6D, S7A. (XLSX)
