# Supplementary figures and images for: The circRNA circVAMP3 restricts influenza A virus replication by interfering with NP and NS1 proteins
Source: PLoS Pathog. 2023 Aug 21;19(8):e1011577. doi: 10.1371/journal.ppat.1011577 (PMC10441791; doi:10.1371/journal.ppat.1011577)

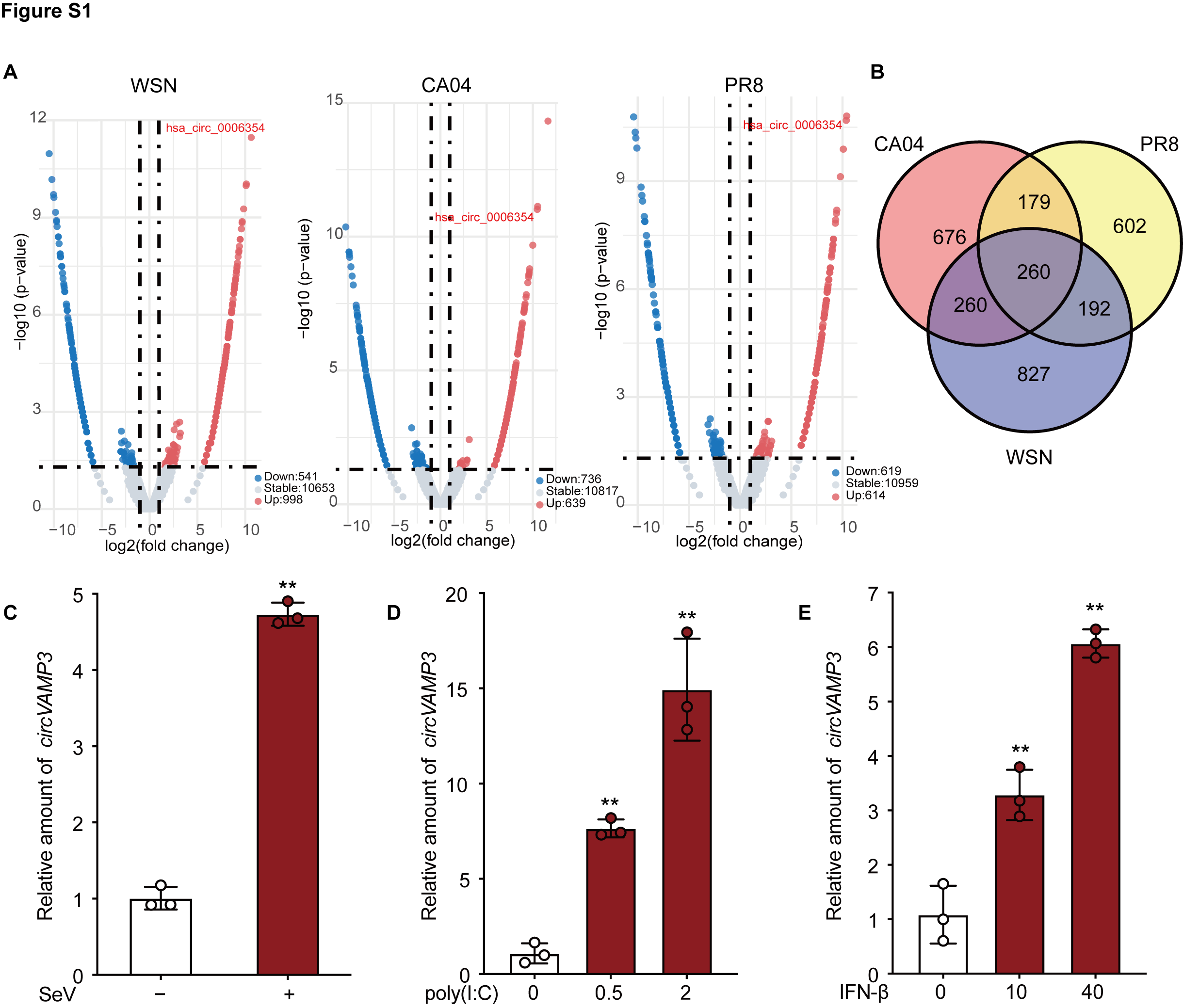

Supplement: S1 Fig — (A, B) A549 cells were infected with IAV (WSN, CA04, or PR8) at an MOI of 1 for 8 h. Total RNA was treated with RNase R and analyzed by RNA sequencing. (A) Volcano plots illustrating differentially expressed circRNAs in WSN-, CA04-, or A/PR8-infected cells compared to mock-infected cells. (B) Venn diagram showing the distribution of differentially expressed circRNAs in IAV-infected cells. The numbers inside the points of intersection indicate the number of common or specific circRNAs. (C, D, E) 293T cells were infected with SeV at 200 HAU/mL for 8 h (n = 3) (C), transfected with poly (I:C) (0.5 μg/mL or 2 μg/mL) for 8 h (n = 3) (D), or treated with IFN-β (10 ng/mL or 40 ng/mL) for 12 h (n = 3) (E), after which the total RNA was extracted, treated with or without RNase R, and quantified using RT-qPCR to detect circVAMP3. Data shown in panels C-E were normalized to GAPDH. Data are presented as the means ± SD. **p < 0.01. (TIF) [file ppat.1011577.s001.tif]

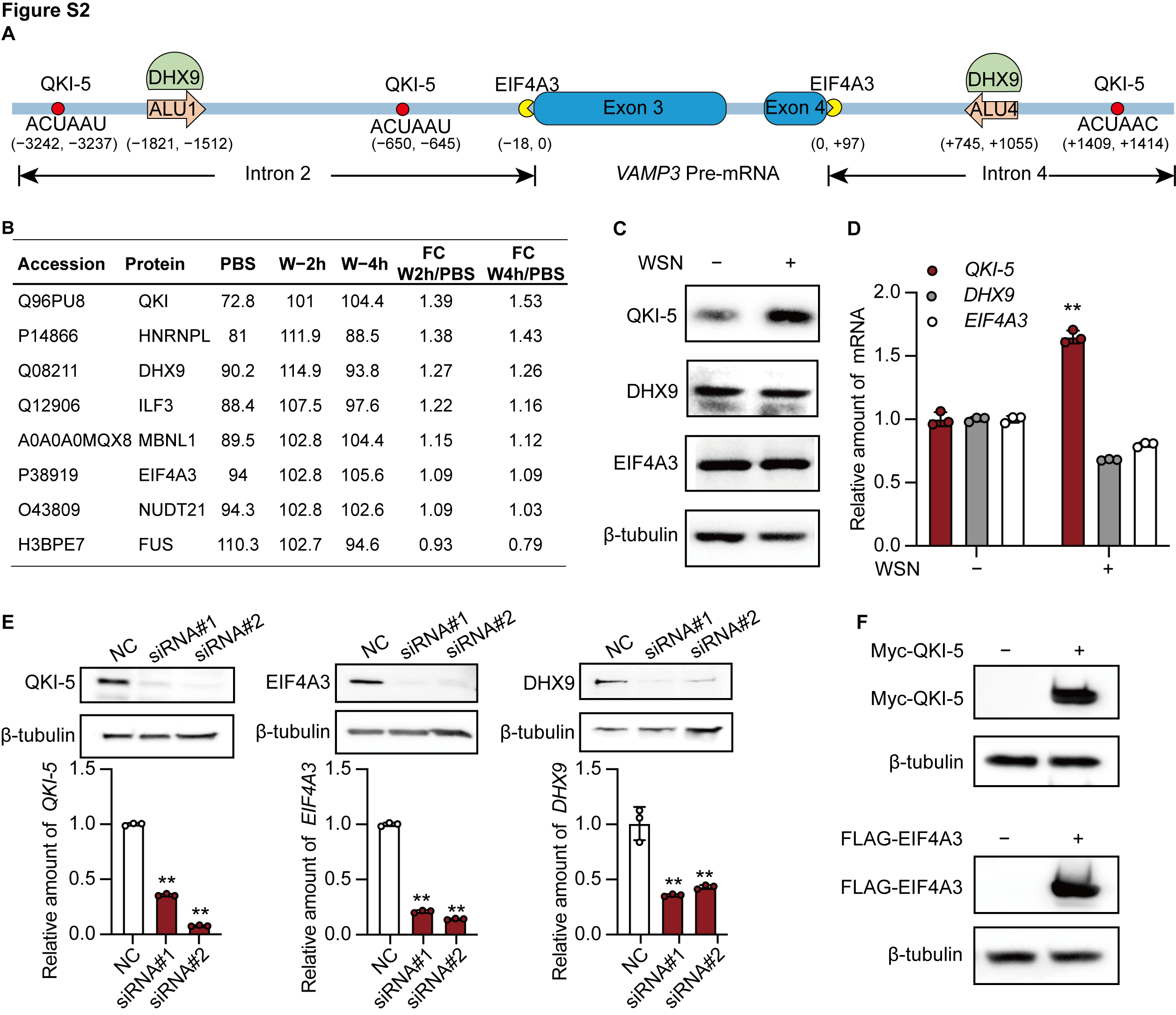

Supplement: S2 Fig — (A) Trans-acting factors, namely, RBPs, promote circRNA biogenesis. (B, C, D) A549 cells were infected with or without WSN at an MOI of 1. (A) The cell lysates were harvested at 2 or 4 h and subjected to quantitative proteomics analysis. Proteins associated with circRNA biogenesis are listed in order of fold change (FC). (B) Schematic illustration of putative binding sites for the three RNA-binding proteins (QKI-5, EIF4A3, and DHX9) in circVAMP3 flanking introns. (C) The cell lysates were collected and subjected to immunoblotting with the indicated antibodies. (D) Total RNA was extracted, and QKI-5, DHX9, and EIF4A3 were quantified using RT-qPCR. (E) siRNA-mediated knockdown of circVAMP3 flanking intron-binding proteins with two independent siRNAs, respectively. Western blots confirmed the knockdown of QKI-5 (E, upper left panel), EIF4A3 (E, upper middle panel), and DHX9 (E, upper right panel). The knockdown efficiency of QKI-5 (E, lower left panel), EIF4A3 (E, lower middle panel), and DHX9 (E, lower right panel) was detected using RT-qPCR. (F) Western blot of overexpressed QKI-5 and EIF4A3. Data are presented as the means ± SD. **p < 0.01. (TIF) [file ppat.1011577.s002.tif]

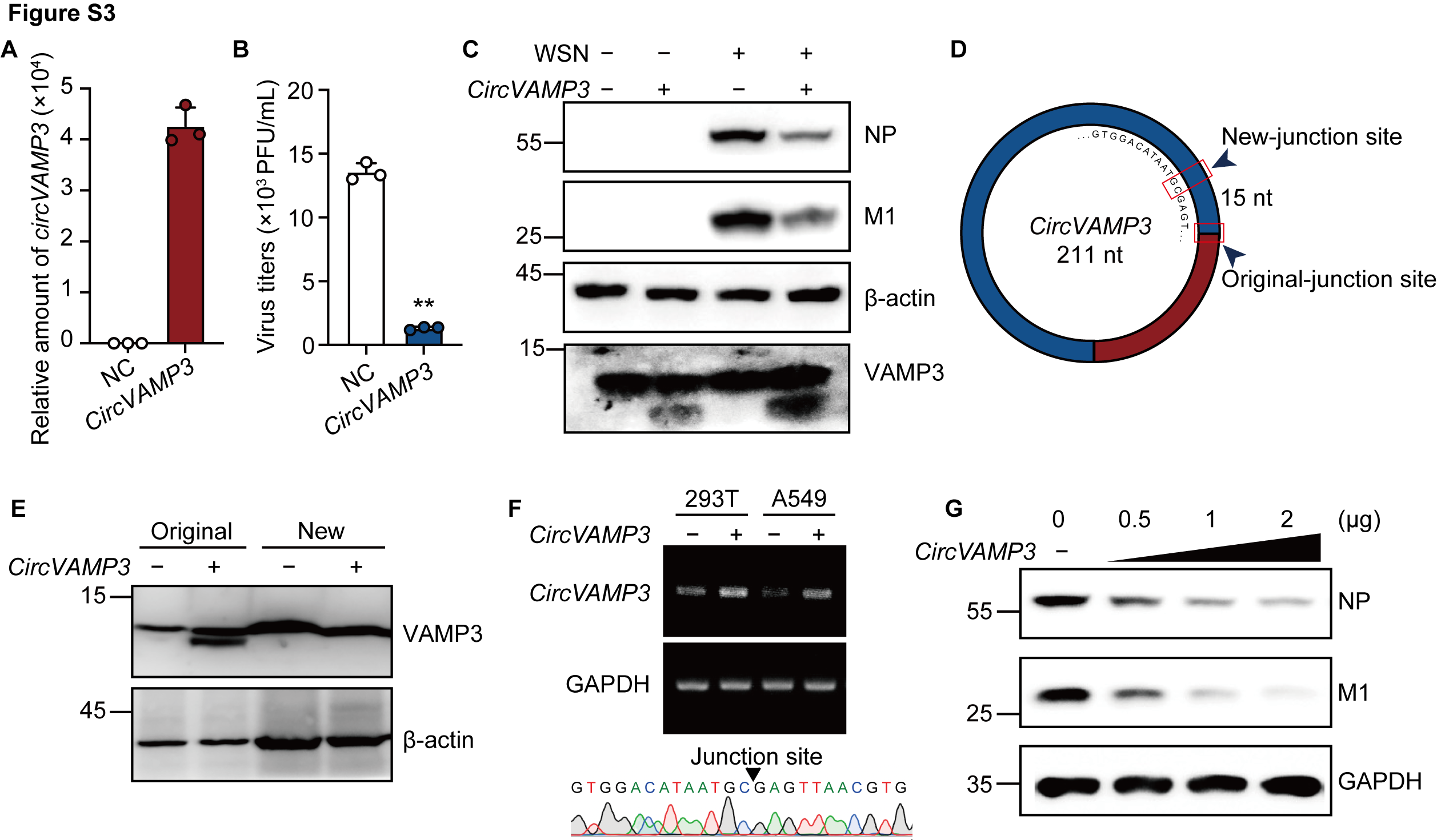

Supplement: S3 Fig — (A) The 293T cells were grown in 6-well plates and transfected with 4 μg circVAMP3 plasmid for 24 h. And then, infected with WSN (MOI = 0.5) for 8 h. The cell supernatants were collected for plaque assays (B), and cell lysates were used for immunoblotting assays (C). (D) The cyclization site of the redesigned circVAMP3. (E) Verification of the product of the circVAMP3 overexpression construct. Original construct, third and fourth exon cyclization; new construct, original junction shifted 15 nt to the third exon. (F, upper panel) Semi-quantification of stably overexpressing cell lines constructed by packaging lentivirus with a new circVAMP3 overexpression vector using reverse primer amplification products separated by agarose gel electrophoresis. (F, lower panel) Sequencing results of single strip cutting in Figure F. (G) The 293T cells in 12-well plates were transfected with a gradient plasmid quantity as indicated in the figure. After 24 h, cells were infected with WSN (MOI = 0.5) for 8 h. The data shown in A and B are presented as the means ± SD; n = 3; **p < 0.01. Data are representative of the results from at least three independent experiments. (TIF) [file ppat.1011577.s003.tif]

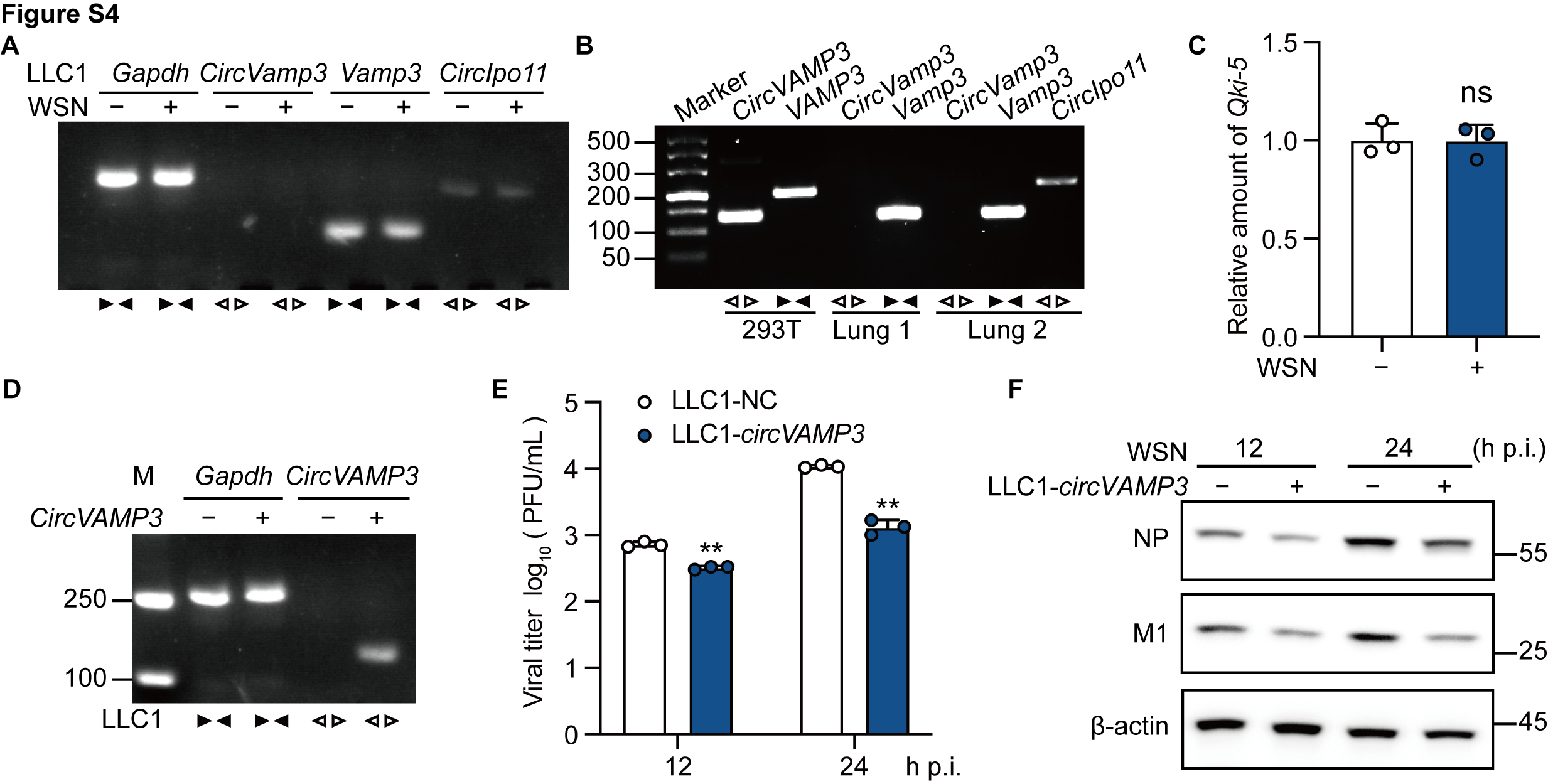

Supplement: S4 Fig — (A) The LLC1 cells were infected with WSN (MOI = 1) for 12 h, followed by RT-qPCR to detect the level of the indicated RNA, using the Vamp3 mRNA and circIpo11 as positive controls. (B) 293T cells and lungs of BALB/c mice were not infected. (C) LLC1 cells were infected with WSN (MOI = 5) or treated with PBS for 4 h. (D) Using lentivirus to infect LLC1 cells. RT-qPCR confirmed that LLC1 cells stably overexpressed circVAMP3. (E and F) The LLC1 cells, stably overexpressed circVAMP3, were infected with WSN (MOI = 0.5) for 12 h and 24 h. The supernatants were collected for plaque assay (E) and the cell lysates were harvested for immunoblotting (F). Data shown in E is presented as the mean ± SD. n = 3; **p < 0.01. These data represent the results of at least three independent experiments. (TIF) [file ppat.1011577.s004.tif]

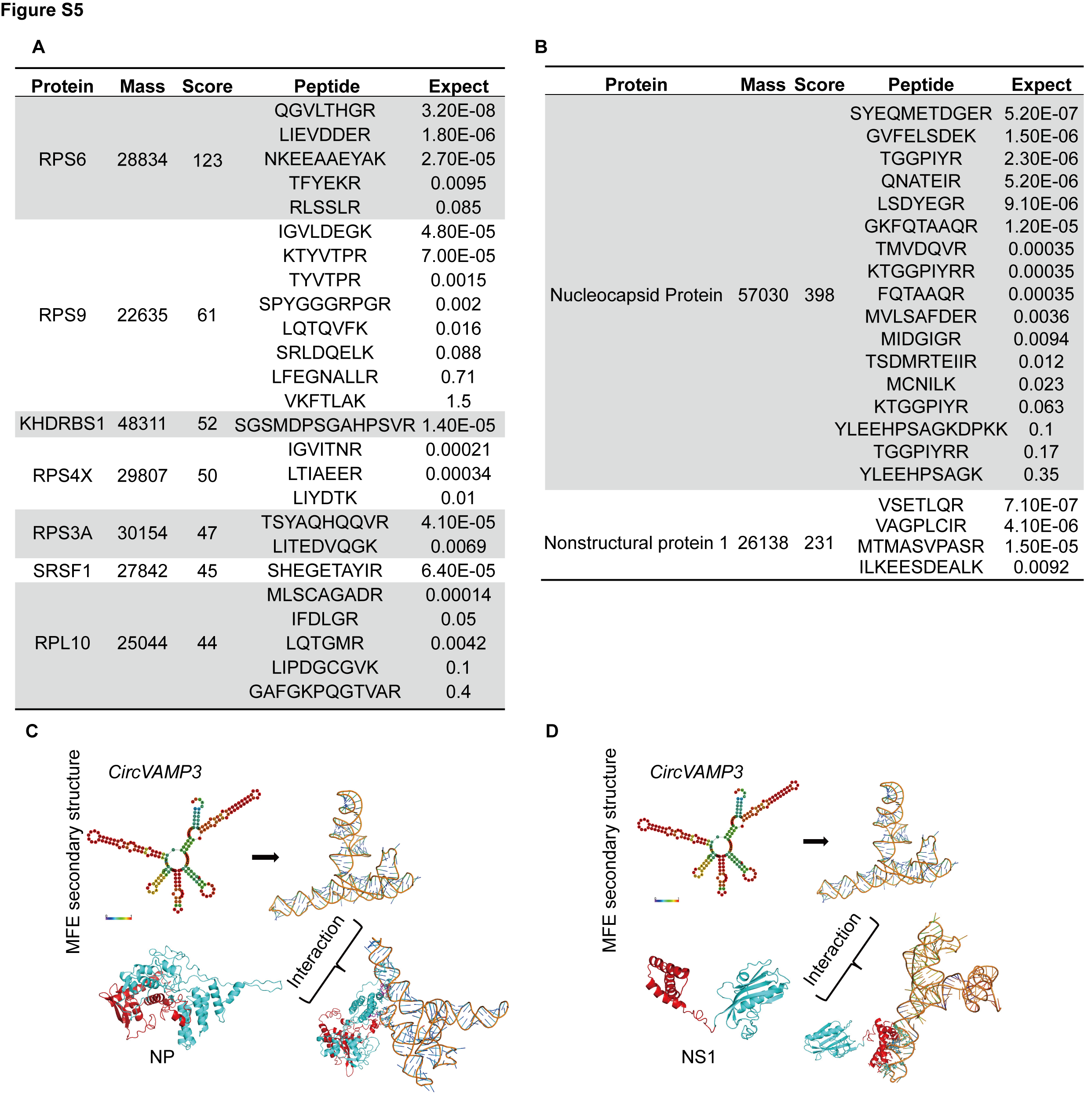

Supplement: S5 Fig — (A, B) A549 cells were infected with WSN. Cell lysates were subjected to a pulldown assay with biotin-labeled circVAMP3 or unlabeled circVAMP3 as the control. The bound proteins were visualized using silver staining. The arrows indicate the bands that were subjected to mass spectrometry analysis. The circVAMP3-bound human proteins (A) and IAV-H1N1 proteins (B) are listed in order of score and expected value. CircVAMP3 was predicted to interact with NP (C) and NS1 (D) by the HADDOCK 2.4 website. The circVAMP3 secondary structure was predicted according to the minimum free energy (MFE). Red indicates strong confidence in the prediction. The 3D structures of NP and NS1 were derived from the I-TASSER server by homology modeling. (TIF) [file ppat.1011577.s005.tif]

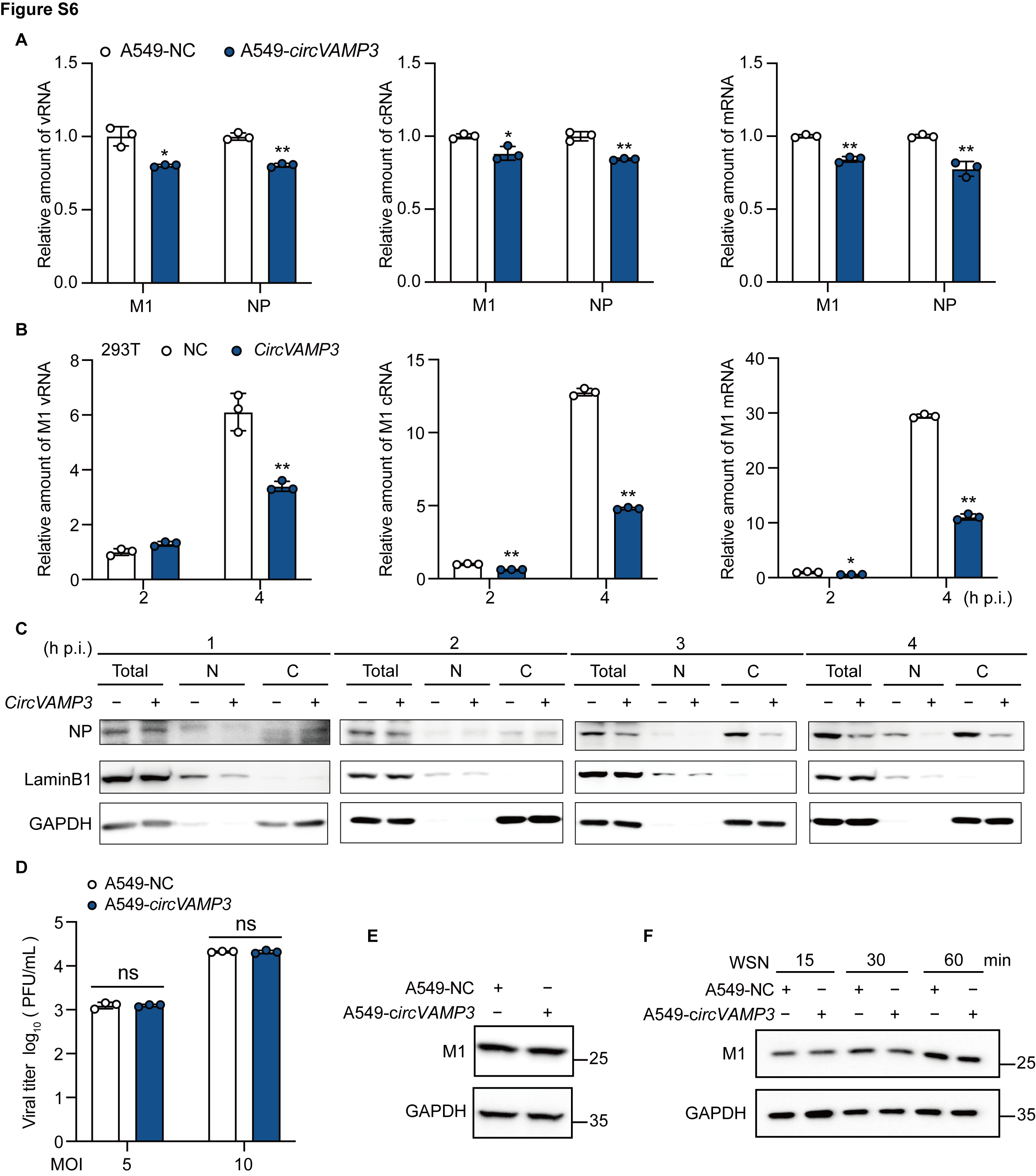

Supplement: S6 Fig — (A) A549 cells stably overexpressing pLC5-circVAMP3 or controls, WSN (MOI = 1) infection and cell lysates were collected at 4 hp.i. for RT-qPCR to detect the level of viral M1 and NP RNAs. (B) The 293T cells were transfected with pLC5-circVAMP3 or pLC5-ciR-GFP for 24 h, followed by being infected with WSN (MOI = 1), and cell lysates were collected at 2 hp.i. and 4 hp.i. for RT-qPCR to detect the level of viral M1 RNAs. (C) Transfected the pLC5-circVAMP3 or pLC5-ciR-GFP into 293T cells at 12-well plates. And then, infected with WSN (MOI = 5), and cell lysate was harvested at the indicated time points. One-tenth of the cell lysate was taken for total protein level detection, and the rest was separated according to the operation instructions. And then, immunoblotted with the indicated antibodies. (D to F) The A549 stable cell lines were infected with WSN on the ice at 4°C for 1 h, followed by a neutral wash (ice-cold PBS = 7.2). E and F MOI = 10. The cells were harvested for plaque assay (D) or immunoblotting (E). (F) After a neutral wash, the cells were cultured with DMEM at 37°C for the indicated time points, followed by an acidic wash (ice-cold PBS-HCl = 1.3) before cell lysis. The internalized WSN was detected by immunoblotting. The data shown in A, B, and D are presented as the means ± SD; n = 3; ns is no significant difference, *p < 0.05, **p < 0.01. (TIF) [file ppat.1011577.s006.tif]

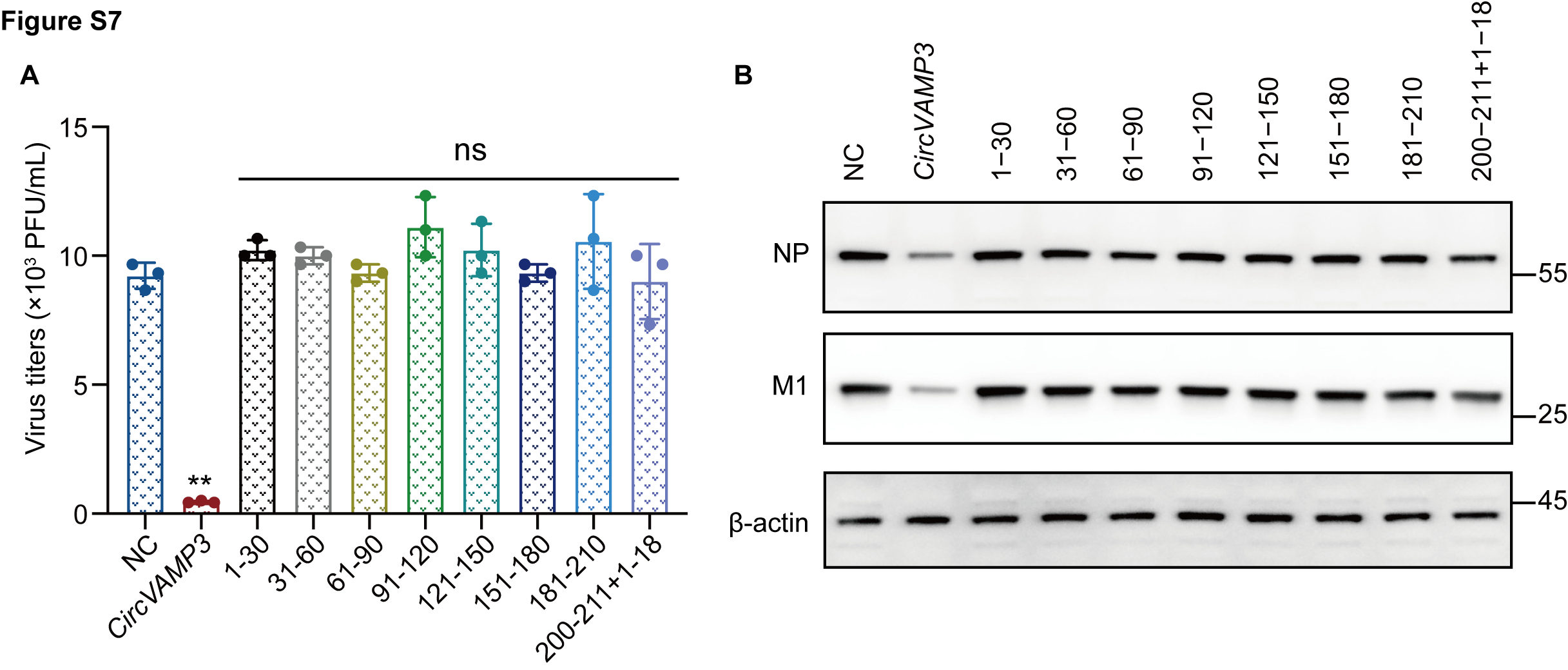

Supplement: S7 Fig — (A and B) The 293T cell were spread into 12-well plates for 16 h, and then transfected with pLC5-circVAMP3 or pLC5-ciR-GFP for 16 h. The rest of the cells were transfected with indicated 100 pmoL RNA segments for 8 h, respectively. Finally, all cells were infected with WSN at an MOI of 0.5 for 12 h. (A) The supernatants of infected cells were collected for plaque tests to determine the virus titer. (B) Cell lysates were collected and used for immunoblotting with corresponding antibodies. (TIF) [file ppat.1011577.s007.tif]
